# Water chlorination and environmental factors are associated with dolphin skin microbiome diversity and composition

**DOI:** 10.3389/fmicb.2026.1934395

**Published:** 2026-10-28

**Authors:** Lorenzo Fiori, Yajuan Lin, Shuai Gu, Guillermo J. Sánchez-Contreras, Krysta Walker, Dara N. Orbach

**Affiliations:** 1 Department of Life Sciences, Texas A&M University-Corpus Christi, Corpus Christi, TX, United States; 2 The Dolphin Company, Cancún, QR, Mexico; 3 Dolphin Quest, Honolulu, HI, United States

**Keywords:** 16S rRNA metabarcoding, common bottlenose dolphins, pH, rough-toothed dolphins, salinity, temperature

## Abstract

Dolphin skin microbiome is constantly exposed to a microorganism-rich medium and represents the first line of defense from environmental stressors and potential pathogens. While some bacteria cause skin diseases and ulcerating wounds, high water temperature, salinity, and pH seem to lower dolphin wound rates and promote accelerated scar healing. Despite its importance in host health, interactions of dolphin skin microbiome with physical and chemical characteristics of habitats remain poorly understood. Using 16S rRNA metabarcoding, we characterized and compared the skin bacteria communities of 87 common bottlenose dolphins (*Tursiops truncatus*) and 4 rough-toothed dolphins (*Steno bredanensis*) living in chlorinated tanks, enclosed lagoons, or open bays. Dolphins living in enclosed lagoons and open bays had skin microbiomes dominated by *Enhydrobacter*, a bacteria genus found on cetacean skin that occurred at low relative abundance in dolphins in chlorinated tanks. The skin microbiome of dolphins in chlorinated tanks was the most diverse and showed a significant increase of *Staphylococcus* relative abundance and prevalence when compared to enclosed lagoons and open bay specimens. Among dolphins sampled in open bays across seasons, summer conditions (higher temperature and pH) were associated with up to 10-fold lower *Mycobacterium* relative abundance, as well as a significant decrease of *Streptococcus* prevalence, genera associated with slow healing of cutaneous wounds in dolphins. Our results reveal novel insights into the association of physical/chemical environmental factors with dolphin skin microbiome composition. We highlight how the relative abundance and prevalence of potentially opportunistic bacteria on dolphins may vary across human-managed and wild habitats, providing crucial information for the improvement of dolphin husbandry practices and conservation.

## 1 Introduction

The skin microbiome is an important component of a host’s defense against pathogens in the external environment (Byrd et al., 2018). As a living physical barrier, microbial communities modulate skin protection from external stressors such as ultraviolet radiation (Patra et al., 2019), contribute to pH acidification (Bullock et al., 2020), maintain optimal moisture levels (Rippke et al., 2002), and promote a host’s lesion repair mechanisms (Canchy et al., 2023). The commensal microorganisms on human skin can produce antimicrobic peptides that prevent colonization by harmful bacteria (Zipperer et al., 2016) and inhibit the growth of potential pathogens by occupying space and consuming resources (Alonzo, 2022).

Skin microbiome composition is associated with various human skin diseases (Khan and Koh, 2024) such as acne (Dessinioti and Katsambas, 2010) and atopic dermatitis (Terada et al., 2006). Changes in skin microbiome composition are linked to diseases in non-human vertebrate species including cattle (Klitgaard et al., 2008), bats (Gargas et al., 2009), frogs (Jani and Briggs, 2014), and trout (Weber et al., 2010). Characterizing skin microbiome can aid conservation efforts for endangered species and improve husbandry practices by providing biomarkers of healthy and diseased individuals (Ross et al., 2019). The identification of commensal bacteria species inhibiting pathogen infection and growth is essential for the development of skin probiotics that can mitigate the population collapse of amphibian species vulnerable to skin diseases (Woodhams et al., 2016). Skin probiotics were successfully used to treat cutaneous lesions in mammals such as rats (Zahedi, 2011), mice (Poutahidis et al., 2013), and horses (Wilmink et al., 2020). Topical application of probiotics may be beneficial for the welfare of human-managed animals and improve the success of rehabilitation and release programs (Kueneman et al., 2016). Given the role of skin microorganism communities in preventing cutaneous infections and promoting wound healing processes, it is crucial to identify disease-associated microorganisms and beneficial commensal bacteria. Furthermore, investigating how environmental factors affect microbiome composition across closely related but different host species may provide valuable insights into host health and management strategies. Cetaceans (whales, dolphins, and porpoises) are fully aquatic mammals that live in a microorganism-rich medium and are exposed to the distinctive physical and chemical characteristics of their habitat waters. Skin microbial communities have been characterized in several baleen and toothed whales (Apprill et al., 2014; Chiarello et al., 2017; Russo et al., 2018; Hooper et al., 2019; Apprill et al., 2020; Robles-Malagamba et al., 2020; Van Cise et al., 2020; Toro et al., 2021; Van Bonn et al., 2022; Zhang, X. et al., 2022; Rhodes et al., 2026), and these communities appear to be species-specific including when multiple species share enclosure water (Chiarello et al., 2017).

Cetacean skin is hairless after early neoteny (Yochem and Stewart, 2009) and directly exposed to abiotic factors (i.e., salinity, temperature, pH). Despite this constant exposure, cetacean skin microbiome composition appears to be distinct from the surrounding water microbiota (Chiarello et al., 2017; Robles-Malagamba et al., 2020; Rhodes et al., 2026; Zhang, X. et al., 2022). The skin microbiome composition of beluga whales (*Delphinapterus leucas*) in managed care facilities remained stable when 50% of habitat water was changed, which likely affected the tank’s bacterial community (Van Bonn et al., 2022). Both host phylogeny and environmental factors shape cetacean skin bacterial communities (Bierlich et al., 2018; Russo et al., 2018; Apprill et al., 2020; Toro et al., 2021; Zhang, X. et al., 2022).

The skin microbiome of cetaceans living in chlorinated aquaria (i.e., treated with sodium hypochlorite) may have a different composition than free-swimming individuals, although this has never been tested. Human skin significantly decreased in microbiome diversity after 1 h of exposure in a chlorinated pool (free chlorine = 2.6 ppm) (Morss-Walton et al., 2022). Some bacteria genera (e.g., *Enterococcus, Pseudomonas, Staphylococcus, Vibrio*) include chlorine-resistant bacteria (CRB) strains able to survive water chlorination and increase up to 200× relative abundance after chlorine treatment (Luo et al., 2021; Wang et al., 2023).

While research on the effects of chlorine on cetacean skin bacterial communities is lacking, other environmental parameters seem to influence epidermal community structure in free-swimming cetaceans. The skin microbiome composition of free-swimming beluga whales varied significantly based on location and season (Van Cise et al., 2020). Water temperature decreases reduced the relative abundance of core bacteria genera on humpback whale (*Megaptera novaeangliae*) skin (Bierlich et al., 2018). The relative abundance of *Psychrobacter* spp., a dominant bacteria genus on the skin of several cetacean and fish species, decreased congruent with water temperatures (Hooper et al., 2019). Coastal common bottlenose dolphins (*Tursiops truncatus*) hosted a less diverse skin microbiome than offshore conspecifics, potentially due to warm water exposure promoting frequent skin sloughing (Russo et al., 2018). Warmer waters may directly alter the dominance of temperature-sensitive bacteria genera and affect the bacteria colonization process by accelerating skin turnover. Despite the importance of skin microbiome as a determinant of host health in cetaceans (Apprill et al., 2014; Sehnal et al., 2021), the effects of physical/chemical environmental parameters other than temperature remain poorly understood.

Exposure to low salinity levels (<10 ppt) can generate skin lesions that compromise the epidermal barrier integrity of common bottlenose dolphins, causing skin microbiome dysbiosis (Ewing et al., 2017; Takeshita et al., 2021). Prolonged exposure to hyposaline waters (<5 ppt) has been linked to freshwater skin diseases in common bottlenose dolphins (Fazioli et al., 2026), Indo-Pacific bottlenose dolphins (*Tursiops aduncus*) and Burrunan dolphins (*Tursiops australis*) (Duignan et al., 2020). In South Texas, USA, skin markings on the dorsal fins of common bottlenose dolphins healed faster among individuals in hypersaline (>35 ppt) compared to adjacent polyhaline (18–30 ppt) bays (Hurst and Orbach, 2022). Skin lesion occurrence rates of dolphins in these same hypersaline and polyhaline bays were highest in winter when temperatures and pHs were low (Guinn et al., 2024). Seasonal variations of environmental factors such as temperature and pH may promote growth of opportunistic pathogenic bacteria and hamper wound healing processes (Bugica et al., 2020). Several bacteria occur in necrotizing and ulcerating cutaneous wounds of stranded dolphins, including *Mycobacterium marinum* (Souter et al., 2026), *Streptococcus iniae* (Souter et al., 2021), and *Vibrio* spp. (Li et al., 2025). While the direct effects of salinity, and pH on cetacean skin microbiome are unknown, research on fish highlight how these factors can shape the epithelial mucus bacterial communities. In wild populations of European eels (*Anguilla anguilla*), increased salinity promoted epithelial mucus microbiome enrichment of *Vibrio* spp. (Carda-Diéguez et al., 2017). A pH shift from 6 to 4 changed the epithelial mucus microbiome taxonomy in Tambaqui (*Colossoma macropomum*) (Sylvain et al., 2016).

Our objective was to compare skin microbiome community composition and diversity across dolphins in different habitats. We aimed to characterize and compare the skin microbiomes of two species of dolphins housed in the same human-managed facility, and of dolphins living in chlorinated aquaria, enclosed lagoons, and open bays. We hypothesized that dolphin skin microbiome compositions and diversity will vary between species (Chiarello et al., 2017; Apprill et al., 2020) and between habitat types. Specifically, we predicted that dolphins in chlorinated tanks will have the lowest skin microbiome diversity (Morss-Walton et al., 2022), and the highest relative abundance of bacteria genera containing CRB strains (Luo et al., 2021; Wang et al., 2023). Among dolphins in open bay systems, we hypothesized that physical and chemical water parameters will affect skin microbiome composition and diversity (Sylvain et al., 2016; Krotman et al., 2020; Hurst and Orbach, 2022; Guinn et al., 2024). We predicted low relative abundances of potentially opportunistic bacteria belonging to genera previously associated with slow-healing wounds (e.g., *Mycobacterium*, *Streptococcus*, *Vibrio*) on the skin of dolphins in open bays during high temperature, pH (Guinn et al., 2024), and salinity exposure (Hurst and Orbach, 2022).

## 2 Materials and methods

### 2.1 Study sites

*Chlorinated Tanks*: The skin of common bottlenose dolphins and rough-toothed dolphins (*Steno bredanensis*) housed in three facilities in Florida (FL1, FL2, FL4) were sampled between October 2023 and February 2024 (Supplementary Table 1). The two dolphin species were housed in separated tanks that shared the same recirculating water. Tank water was sourced from adjacent marine bays and treated with sodium hypochlorite to maintain a total chlorine concentration at the threshold of 1 mg/L (free chlorine = 0.8 ppm). Facilities were equipped with a filter system circulating water through different pools.

*Enclosed Lagoons*: Common bottlenose dolphins housed in three facilities were sampled between December 2023 and September 2024. One enclosed lagoon was located in Florida (FL3), and two were an open system ocean fed lagoon with tidal fluctuations (HI1) and a semi open system ocean fed lagoon (HI2) in different locations in Hawaii, USA (Supplementary Table 2). Water was actively pumped (HI1, HI2) or flowed (FL3) into the habitats from the ocean and was untreated.

*Open Bays*: Free-swimming common bottlenose dolphins were remote biopsy sampled in two open bay systems in South Texas, USA in May and August 2024 (Supplementary Table 3).

Redfish Bay (RB) is polyhaline while Upper Laguna Madre (ULM) is hypersaline.

### 2.2 Microbiome sample collection

Skin microbiome samples were collected from dolphins in managed care facilities. There was a maximum of three attempted sample collections per dolphin. Sampling effort was halted immediately if dolphins moved away and/or showed signs of discomfort. Sampled dolphins were in healthy condition and did not receive antibiotics.

The ventrum of a dolphin was dried with sterile gauze as it voluntarily remained stationary in dorsal recumbency by the perimeter of the enclosure. A sterile 15.24 cm long cotton swab (Dukal, Ronkonkoma, NY, USA) was vigorously rubbed on a dolphin’s ventrum >20 cm cranial to the genital slit. The swab handle was cut ~2.5 cm from the tip and placed with the cotton applicator tip facing down into a cryovial prefilled with 1 mL of RNAlater (Thermo Fisher Scientific, Waltham, MA, USA). The cryovial was inverted gently to mix the medium and the sample was promptly placed at −20 °C until dry ice shipment in the same day to the processing facilities at Texas A&M University-Corpus Christi where it was stored at −80 °C.

Skin samples were collected from free-swimming common bottlenose dolphins during dedicated boat-based surveys (6 m Inmar rigid hull inflatable boat, 150 hp. outboard engine). Skin and blubber samples were collected by remote biopsy using a crossbow (Barnett Panzer V, 68 kg draw weight, Barnett Outdoors, LLC, Tarpon Springs, FL USA) and modified darts (10 × 25 mm stainless-steel; Ceta-Dart, Copenhagen, Denmark) with prongs to hold tissue and foam to facilitate ejection upon contact and floatation. Swabbing biopsies from dolphins yields DNA sufficient to characterize skin microbiome (Hansen et al., 2026). All biopsy instruments were sterilized prior to sample collection following a standardized protocol (Sinclair et al., 2015). Only dolphins that appeared to be in healthy condition and were at least 1 year old were sampled. A maximum of three biopsy attempts were made for any dolphin group. Darts were deployed at an approximate distance of 3–7 m from the target dolphin, aiming 7–10 cm below the dorsal fin. Upon ejection from the dolphin’s body, the darts were immediately retrieved, and the skin was swabbed and stored using the same procedure described for dolphins in managed care facilities. Although no significant differences were found in the skin microbiomes of common bottlenose dolphins sampled at different body areas (Chiarello et al., 2017), we acknowledged that sampling the skin of dolphins under human care and in the wild at different body sites may introduce an artifact. Swabs were placed in liquid nitrogen within 5 min of dart deployment. Photographs of dolphin dorsal fins were taken with a Sony Cyber-Shot RX10 IV (Sony, Minato-ku, Tokyo, Japan) to identify each dolphin based on individually distinct dorsal fin markings (Würsig and Würsig, 1977), prevent resampling the same individual, and ensure the sample was collected in an approximately consistent location of the dolphin. Dolphin groups were monitored for behavioral responses to the biopsy dart deployment (e.g., tail slap, breach) and were monitored for at least 15 min after deployment to ensure they resumed typical behaviors.

### 2.3 Environmental data collection

A water sample and environmental parameter data were collected immediately after skin swabbing at the same location. A 1 L sterile sealable glass bottle was submerged ~1 m deep, opened, filled with water, and resealed while submerged. A 60 mL luer-lock syringe was filled with the collected water and pushed through a Sterivex™ 0.22 μm filter unit (MilliporeSigma, Burlington, MA, USA). The process of pushing water through the filter was repeated until resistance occurred. The amount of water filtered was recorded. The filter cartridge was sealed on both ends with Loctite putty and kept in liquid nitrogen or −20 °C until reaching the processing facilities at Texas A&M University-Corpus Christi where it was stored at −80 °C.

Data on temperature and pH were collected in managed care facilities using a HI2020-012 edge® Multiparameter pH Meter (Hanna® Instruments, Woonsocket, RI, USA). Salinity was measured with an RSA-BR32T refractometer (Cole-Parmer, Vernon Hills, IL, USA) in both managed care facilities and open bay systems. In open bay systems, water temperature was measured with a calibrated ProSolo kit (YSI, Yellow Springs, OH, USA), and pH was measured using a calibrated Ecosense® pH 10A (YSI, Yellow Springs, OH, USA).

### 2.4 Bacterial DNA extraction and purification

RNAlater solution (~1 mL) concentration was performed for each swab sample using Amicon® Ultra centrifugal filters 10KDa (MilliporeSigma, Burlington, MA, USA): two 30 min centrifuge rounds at 14000 rpm for each specimen were conducted. The concentrated solution and the swab were then processed together. ATL buffer (360 μL) was added to the samples. Bacterial cell mechanical rupture was performed by bead-beating for 1 min at a frequency of 2,520 rpm in a mini-bead beater with 0.1 mm zirconium beads (BioSpec Products, Bartlesville, OK, USA). DNA Mini kit (Qiagen, Hilden, Germany) swab and tissue protocols were used for DNA extractions. DNeasy Plant Mini kit (Qiagen, Hilden, Germany) was used for Sterivex™ filters. Qubit™ 3.0 fluorometer and Qubit™ dsDNA HS Assay Kit (Thermo Fisher Scientific, Waltham, MA, USA) were used to quantify purified DNA concentrations. Extracted DNA was stored at −80 °C.

### 2.5 DNA amplification and indexing

Metabarcoding libraries were prepared for sequencing on MiSeq platform (Illumina, San Diego, CA, USA). Forward and reverse fusion PCR primers targeting the hypervariable V3-V4 region (~460 bp) (Herlemann et al., 2011) of the 16S rRNA gene were used. The forward primer sequence was 5′-CCTACGGGNGGCWGCAG-3′ and the reverse primer sequence was 5′-GACTACHVGGGTATCTAATCC-3′. Fusion primers included a heterogeneous spacer up to 4 nucleotide long, a 6 nucleotide long index, and the Illumina MiSeq sequencing adapter (Fadrosh et al., 2014). Indexing PCR reactions followed an established protocol (Robles-Malagamba et al., 2020). PCR mix contained 0.175 μL of Platinum Taq DNA Polymerase (Thermo Fisher Scientific, Waltham, MA, USA), 3.5 μL of 10x PCR buffer, 1.4 μL of 50 mM MgCl2, 0.7 μL of 10 mM dNTPs, 1.75 μL of 1 μM of forward and reverse primer, 20.5 μL of molecular grade water, and 5.25 μL of sample. PCR reactions initiated with 3 min denaturation at 95 °C. Cycles (30) included denaturation at 95 °C for 30 s, annealing at 55 °C for 30 s, and extension at 72 °C for 30 s. The final elongation step was conducted at 72 °C for 5 min. Amplification success was assessed by gel electrophoresis. Samples corresponding to a very weak or completely absent band around 460 bp were discarded and indexing PCR was repeated increasing the number of cycles to 35. PCR amplicons were purified using Mag-Bind® Total Pure NGS (Omega Bio-Tek, Norcross, GA, USA). Amplicon concentrations were quantified using AccuBlue® High Sensitivity dsDNA Quantification Solution (Biotium, Fremont, CA, USA). Equimolar pools were sequenced on an Illumina MiSeq platform using a 2 × 300-cycle v3 MiSeq Reagent Kit (Illumina, San Diego, CA, USA) at Texas A&M AgriLife Genomics & Bioinformatics Service. In total, four MiSeq 300PE runs were conducted for 136 amplified samples.

### 2.6 Bioinformatic analysis

All raw sequencing data were processed in QIIME2 (version 2024.2) (Bolyen et al., 2019). Paired-end reads were demultiplexed using sample-specific forward and reverse barcodes, and primers were removed with cutadapt plugin (Martin, 2011). Denoising was performed with the dada2 denoise-paired plugin (Callahan et al., 2016), with truncation lengths of 244–272 bp for forward reads and 182–198 bp for reverse reads depending on the run, while all other parameters were kept at default settings. This process simultaneously filtered low-quality reads, merged paired-end sequences, and removed chimeras. Truncation positions were selected independently for each of the four sequencing runs from the demultiplexed quality profiles, retaining bases while the median Phred score remained at or above 35 and preserving at least 12 bp of overlap for read merging. The exact values per run, the resulting merged amplicon lengths measured from the denoised sequences, read retention through each processing step, and the per-run quality profiles with truncation positions marked are provided as Supplementary Figure 1. A total of 9,121,595 non-chimeric 16S ribosomal RNA (rRNA) V3-V4 region reads were included in the bacterial community characterization. Representative amplicon sequence variant (ASV) sequences, feature tables, and denoising statistics were generated for downstream analysis.

Taxonomic classification of ASVs occurred using the QIIME2 feature-classifier classify-sklearn method. A naïve Bayes classifier was trained with the fit-classifier-naive-bayes function on the SILVA 138.2 reference database (Quast et al., 2012; Yilmaz et al., 2014; Glöckner et al., 2017), trimmed to the targeted region. ASVs assigned to mitochondria and chloroplasts were removed from the feature table prior to downstream analyses.

Alpha diversity metrics (observed features, Shannon, Simpson, Pielou’s evenness, Chao1) were calculated in QIIME2 after rarefaction to an even sequencing depth of 10,000 reads per sample, determined from rarefaction curve inspection. Samples below this threshold were excluded, leaving 91 skin samples; the lowest-depth sample retained contained 20,532 reads.

### 2.7 Statistical analysis

Differences in alpha diversity amongst habitat types (tank, enclosed lagoon, open bay) were tested per index with Kruskal-Wallis rank-sum test (Kruskal and Wallis, 1952), followed by pairwise Wilcoxon rank-sum tests (Wilcoxon, 1945) with Benjamini-Hochberg (FDR) correction (Benjamini and Hochberg, 1995). Differences in overall community composition were tested with Non-metric multidimensional scaling (NMDS) and PERMANOVA (9,999 permutations) on Bray–Curtis dissimilarity (Bray and Curtis, 1957) and Jaccard distance, calculated on the rarefied feature table. Overlap between skin and water communities was quantified separately for each habitat type at both sequence-variant and genus level, counting a taxon as present when detected in at least two samples. In addition to the number of shared taxa, the proportion of skin reads belonging to shared taxa was calculated. The potential effect of age, sex, and duration of housing on microbiome variation was tested for common bottlenose dolphins under human care (*n* = 58) as age and sex were not available for free-swimming dolphins. Each covariate was tested alone and again with facility fitted first, using PERMANOVA with 9,999 permutations, distance-based redundancy analysis conditioned on facility, and variance partitioning. Homogeneity of multivariate dispersion was assessed by PERMDISP (betadisper with permutest). Pairwise dispersion differences were tested with Tukey’s HSD. Sequencing run was fitted before habitat type to assess batch effects. As dolphins at one facility share water, air, diet, and care, the habitat effect was also tested with sampling site as the unit of permutation, enumerating all 560 assignments of habitat labels to the eight sites to obtain an exact p value.

Mean relative abundance (r.a.) and prevalence (*p*) of fifteen bacteria genera containing CRB strains (*Acinetobacter, Aeromonas, Bacillus, Citrobacter, Enterococcus, Halomonas, Klebsiella, Legionella, Methylobacterium, Mycobacterium, Pseudomonas, Sphingomonas, Staphylococcus, Stenotrophomonas, Vibrio*) were compared between chlorinated tank, enclosed lagoon, and open bay dolphin and water samples. Counts were centered log-ratio (CLR) transformed (Aitchison, 1982; Gloor et al., 2017) after adding a pseudo-count corresponding to half of the minimum non-zero value in the dataset (Mallick et al., 2021). CLR transformed-counts were compared using Kruskal-Wallis rank-sum test (Kruskal and Wallis, 1952), followed by pairwise Dunn tests (Dunn, 1964) with Benjamini-Hochberg (FDR) correction (Benjamini and Hochberg, 1995). To optimize statistical power and avoid the artifacts of extreme zero-inflation on rank-based testing, bacterial genera with a prevalence <30% were excluded from the analysis (Weiss et al., 2017; Cao et al., 2020). Prevalence was calculated dividing the number of samples positive for a bacteria genus by the total number of dolphins in a particular habitat type. Chi-square (Pearson, 1900) was used to compare prevalence between the three habitat types, while Fisher–Freeman–Halton exact test (Freeman and Halton, 1951) was used when expected cell counts were <5. Fisher’s exact test (Fisher, 1922) with Bonferroni correction (Dunn, 1961) was used for pairwise comparisons. Bacteria genera containing CRB strains were selected based on existing literature demonstrating chlorine-resistance (Luo et al., 2021; Wang et al., 2023). The criteria to define CRB strains is a 200× increase in relative abundance after chlorine disinfection treatment and existing evaluation methods compare logarithmic inactivation rates, concentration-time values, minimal inhibitory concentration, and/or survival rate (Luo et al., 2021). CRB genera that were not detected in dolphin skin swabs and habitat water samples were excluded from the analysis.

Canonical correspondence analysis (CCA) was performed in R (vegan package) (Oksanen et al., 2026) constraining skin microbial community composition to environmental variables (temperature, pH, salinity). Community data were relative frequency-transformed prior to ordination. We applied permutation-based stepwise model selection with forward and backward steps and 999 permutations to identify which environmental variables were significantly associated with community composition. Each retained variable’s significance was assessed by permutation test, and collinearity among retained variables was checked via variance inflation factors (VIF). All three variables are displayed as biplot vectors; pH, which was not retained by the selection procedure, is shown for comparison as nearly collinear with temperature. PERMANOVA, PERMDISP, NMDS, distance-based redundancy analysis, variance partitioning, and CCA were performed with the vegan package in R (Oksanen et al., 2026). Data handling, remaining statistics, and figure generation used Python (NumPy, pandas, SciPy, Matplotlib) (Harris et al., 2020; Virtanen et al., 2020; Hunter, 2007).

Mean relative abundance and prevalence of potential opportunistic pathogens associated with slow skin healing (e.g., *Mycobacterium*, *Streptococcus, Vibrio*) were calculated and compared between free-swimming dolphins sampled in RB and ULM in Spring and Summer using the same methods described for bacteria genera containing CRB strains.

## 3 Results

### 3.1 Environmental parameters

Chlorinated tanks had similar environmental conditions across facilities, including temperatures (23–25 °C), salinities (33–35 ppt), and pH (7.4–7.5) (Supplementary Table 1). The semi-enclosed lagoons had temperatures ranging between 21.7 (FL3) and 27.2 °C (HI1, HI2) and were polyhaline (HI1: 30 ppt, HI2: 24–25 ppt). pH ranged between 7.7 (HI2) and 8.3 (HI1, FL3) (Supplementary Table 2). Open bay systems were characterized by seasonal variations in temperature (Spring: 26.2–27.6 °C; Summer: 30.7-32.6 °C) and pH (Spring: 7.6–8.1; Summer: 8.3–8.8). Salinity varied by location (RB: 20–28 ppt; ULM: 29–45 ppt) and season for RB (Spring: 20–23 ppt; Summer: 25–28 ppt), while it decreased for ULM (Spring: 36-45 ppt; Summer: 29-39 ppt) (Supplementary Table 3).

### 3.2 Microbiome characterization

The skin microbiomes were characterized for 38 common bottlenose dolphins and 4 rough-toothed dolphins housed in chlorinated tanks, 20 common bottlenose dolphins living in enclosed lagoons, and 29 free-swimming common bottlenose dolphins inhabiting two open bay systems (n_RB_ = 15; n_ULM_ = 14), and 45 water samples (n_tank_ = 11; n_enclosed lagoon_ = 6; n_open bay_ = 28) (Figure 1). Two samples from common bottlenose dolphins living in a chlorinated tank (ID = 26; Supplementary Table 1) and ULM (ID: 35B; Supplementary Table 3) did not yield a sufficient number of reads (*n* < 10,000) and were excluded from microbiome characterization. The lowest-depth sample among the remaining 91 samples contained 20,532 reads, so no sample was close to the rarefaction threshold. Rarefaction curves for observed features, Shannon diversity and Chao1, are provided as Supplementary File 1. A total of 20,501 representative amplicon sequence variants (ASV) were identified from the V3-V4 region of 16S rRNA gene.

**Figure 1:**
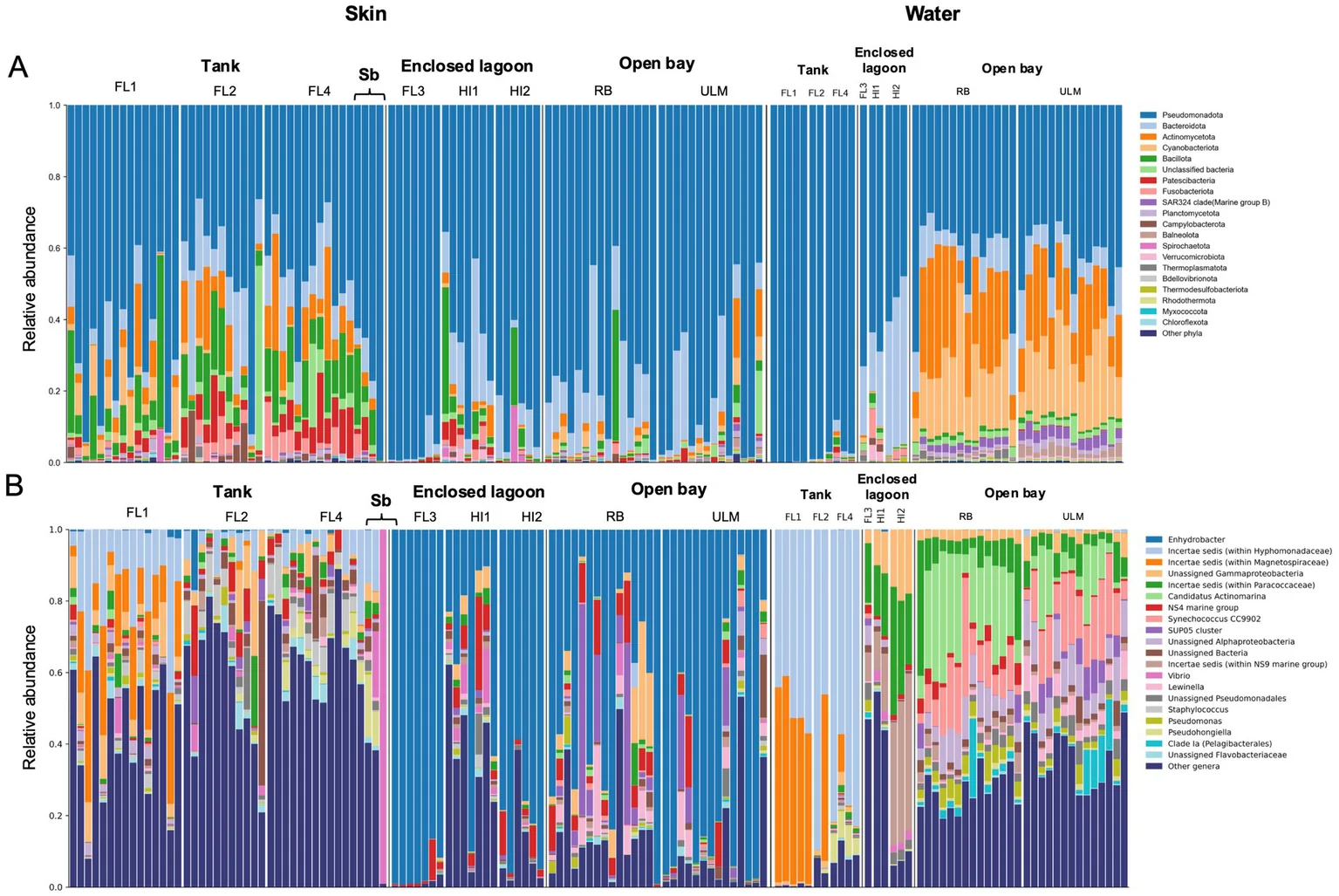
Taxonomic composition of dolphin-associated microbiomes. The stacked bar chart shows the relative abundance of **(A)** bacterial phyla and **(B)** genera for individual 16S rRNA gene amplicon samples. Common bottlenose dolphins (*Tursiops truncatus*, *n* = 87) are grouped by facility (FL1–FL4, HI1, HI2) and open bay system (RB, ULM). All samples from rough-toothed dolphins (*Steno bredanensis*, Sb; *n* = 4) were collected from one facility (FL4). The water samples (*n* = 45) were collected simultaneously with the skin tissue samples. Each vertical bar represents one sample, with the corresponding source annotated above.

Batch effects were assessed in three ways. Sequencing run was included as a term in PERMANOVA and fitted before habitat type; habitat type remained significant, explaining a further 10.3% of variation in Bray-Curtis distances and 5.2% in Jaccard distances (both *p* < 0.001). Multivariate dispersion did not differ among runs (PERMDISP *p* = 0.58). Because sequencing run and sampling site are partially confounded by the study design, sequencing run effects were additionally tested within FL4, the only facility whose samples span three runs; no effect was detected on Bray-Curtis distances (*p* = 0.37) and only a marginal effect on Jaccard distances (*p* = 0.055).

### 3.3 Dolphin species comparisons

Bray–Curtis dissimilarity and Jaccard distance showed that the skin microbiome compositions of rough-toothed dolphins (*n* = 4) differed significantly (Bray-Curtis PERMANOVA: *R*^2^ = 0.14, *p* = 0.004; Jaccard PERMANOVA: *R*^2^ = 0.09, *p* < 0.001) from common bottlenose dolphins (*n* = 12) inhabiting the same facility (Supplementary Figure 2). PERMDISP was non-significant for Bray–Curtis dissimilarity (*p* = 0.147), indicating a difference in composition rather than dispersion, but significant for Jaccard distance (*p* = 0.013), suggesting that part of the presence/absence signal derives from spread. One rough-toothed dolphin (ID = 50; Supplementary Table 1) skin microbiome was almost entirely composed of two ASVs (*Vibrio alginolyticus,* r.a. = 50.9%; *Vibrio parahaemolyticus,* r.a. = 48.1%). While both *Vibrio* species have been associated with severe cutaneous wound infections in dolphins (Li et al., 2025; Souter et al., 2026), the rough-toothed dolphin did not show signs of skin disease. *Vibrio* spp. were not dominant in the tank water (r.a. = 0.2%) nor detected in extraction blanks.

At the phylum level, rough-toothed dolphin skin microbiome was characterized by Pseudomonadota (76.0%), Bacillota (9.1%), Bacteroidota (4.8%), Patescibacteria (2.9%), Actinomycetota (1.9%), and Fusobacteriota (1.7%). The skin microbiome of common bottlenose dolphins had lower mean r.a. of Pseudomonadota (45.9%), and higher mean r.a. of Bacteroidota (11.3%), Patescibacteria (8.1%), Actinomycetota (11.9%), and Fusobacteriota (4.2%). Genera with the highest mean r.a. and *p* in both dolphin species included *Pseudohongiella* (r.a._Sb_ = 8.2%, p_Sb_ = 100%; r.a._Tt_ = 2.8%, p_Tt_ = 100%) and *Dongia* (r.a._Sb_ = 1.5%, p_Sb_ = 75%; r.a._Tt_ = 1.2%, p_Tt_ = 83%). *Vibrio* was more abundant in rough-toothed dolphins (r.a._Sb_ = 29.5%, p_Sb_ = 100%; r.a._Tt_ = 3.0%, p_Tt_ = 42%) while *Aggregatibacter* was more dominant in common bottlenose dolphins (r.a._Sb_ = 1.2%, p_Sb_ = 100%; r.a._Tt_ = 7.4%, p_Tt_ = 100%).

### 3.4 Habitat type comparisons

Regardless of habitat type, common bottlenose dolphins had similar Chao 1 richness (*χ*^2^_df = 2_ = 2.37, *p* = 0.305; Figure 2A). Contrary to predictions, the skin microbiome composition of dolphins housed in chlorinated tanks showed the highest Shannon diversity (*χ*^2^_df = 2_ = 24.74, adjusted *p* < 0.001; Figure 2B), that seemed mostly driven by a significantly higher Pielou evenness (*χ*^2^_df = 2_ = 30.12, adjusted *p* < 0.001; Figure 2C).

**Figure 2:**
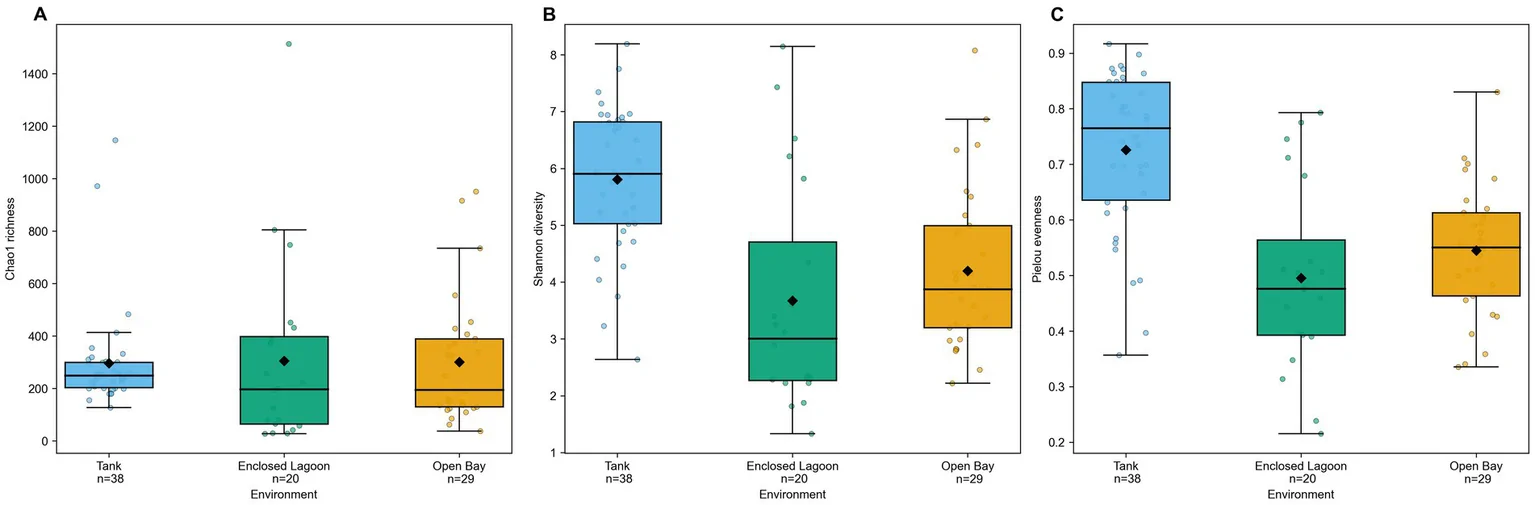
Alpha diversity of dolphin-associated skin microbiomes for each habitat type. Box-and-whisker plots of: **(A)** Chao1 richness, **(B)** Shannon diversity, and **(C)** Pielou’s evenness for common bottlenose dolphin (*Tursiops truncatus*)-associated microbiomes for chlorinated tanks (*n* = 38), enclosed lagoons (*n* = 20), and open bays (*n* = 29). Each data point represents an individual 16S rRNA amplicon sample. Boxes show the median and interquartile range (IQR), whiskers extend to 1.5 × IQR. Black diamonds indicate group means. Sample sizes (*n*) for each habitat type are on the *x*-axis.

Skin microbiome communities were distinct from the surrounding water (Figure 1 and Supplementary Figure 3). At the genus level, 82.2% of skin reads in chlorinated tanks, 93.2% in enclosed lagoons and 97.6% in open bays belonged to genera also present in the corresponding water (Supplementary Figure 3A). At the sequence-variant level the equivalent proportions were 44.2, 38.4 and 49.6% (Supplementary Figure 3B). Therefore, skin communities shared most of their genera with the surrounding water but comprised largely distinct sequence variants within those genera, with roughly half to two-thirds of skin reads belonging to variants not detected in the corresponding water samples. The overlap was markedly asymmetric indicating that dolphin skin carried an abundant component absent from the water column. While skin and water sample numbers were unequal in tanks (42 vs. 11) and enclosed lagoons (20 vs. 6), which inflated the skin-exclusive counts, the open-bay comparison was balanced (29 vs. 28) and confirmed the proportions mentioned above. Skin microbiome composition clustered by habitat type (Bray-Curtis PERMANOVA: *R*^2^ = 0.19, *p* < 0.001; Jaccard PERMANOVA: *R*^2^ = 0.09, *p* < 0.001), with skin samples from dolphins in chlorinated tanks most distinct from other habitats (Figure 3). Skin bacterial communities clustered by facility (Bray-Curtis PERMANOVA: *R*^2^ = 0.41, *p* < 0.001; Jaccard PERMANOVA: *R*^2^ = 0.21, *p* < 0.001). Dispersion differed significantly among habitat types on both distance measures (Bray-Curtis *F*(2,84) = 7.60, *p* < 0.001; Jaccard *F*(2,84) = 7.39, *p* < 0.001), with chlorinated tanks showing the greatest dispersion and open bays the least; tank–open bay contrast was significant in pairwise comparison (Tukey *p* = 0.0006). Dolphins housed at the same facility share water, air, diet and veterinary care and are not independent replicates with respect to habitat type. Habitat test was therefore repeated using sampling site as the unit of permutation, enumerating all 560 possible assignments of habitat labels to the eight sites to obtain an exact *p* value. Habitat type remained significant (*p* = 0.032 for Bray-Curtis, *p* = 0.004 for Jaccard). Age and duration of housing were fitted in separate models as they were almost perfectly collinear (Pearson *r* = 0.988). Facility explained 43.5% of variation in skin microbiome composition (*p* < 0.001). Once facility was accounted for, age and duration in human care were not significant; variance partitioning attributed 37.8% of variation to facility independently, 0.09% to age and 0.07% to duration in human care. Sex was marginal after facility in PERMANOVA (*p* = 0.067) yet significant when conditioned on facility in dbRDA (Bray–Curtis *p* = 20.022; Jaccard *p* = 0.24). No covariate was associated with alpha diversity after adjusting for facility and correcting for multiple comparisons. At the phylum level, the skin microbiome of dolphins in chlorinated tanks was characterized by Pseudomonadota (54.0%), Bacillota (10.5%), Bacteroidota (10.4%), Actinomycetota (8.0%), Patescibacteria (4.6%), Cyanobacteriota (3.1%), and Fusobacteriota (2.5%). Dolphins in enclosed lagoons and open bays had bacterial community compositions dominated by Pseudomonadota (r.a._enclosed lagoon_ = 81.1%; r.a._open bay_ = 78.4%), Bacteroidota (r.a._enclosed lagoon_ = 8.7%; r.a._open bay_ = 12.1%), Bacillota (r.a._enclosed lagoon_ = 4.1%; r.a._open bay_ = 1.9%), and Actinomycetota (r.a._enclosed lagoon_ = 1.0%; r.a._open bay_ = 2.1%). *Enhydrobacter* genus had a low relative abundance among dolphins in chlorinated tanks (r.a._tank_ = 0.9%, p_tank_ = 92.1%), and was dominant in dolphins from other habitats (r.a._enclosed lagoon_ = 70.7%, p_enclosed lagoon_ = 100%; r.a._open bay_ = 58.4%, p_open bay_ = 100%). Similarly, *Enhydrobacter* had the lowest mean relative abundance in tank water samples (r.a._tank_ = 0.0171%, p_tank_ = 36.4%; r.a._enclosed lagoon_ = 0.6%, p_enclosed lagoon_ = 100%; r.a._open bay_ = 0.0256%, p_open bay_ = 50.0%).

**Figure 3:**
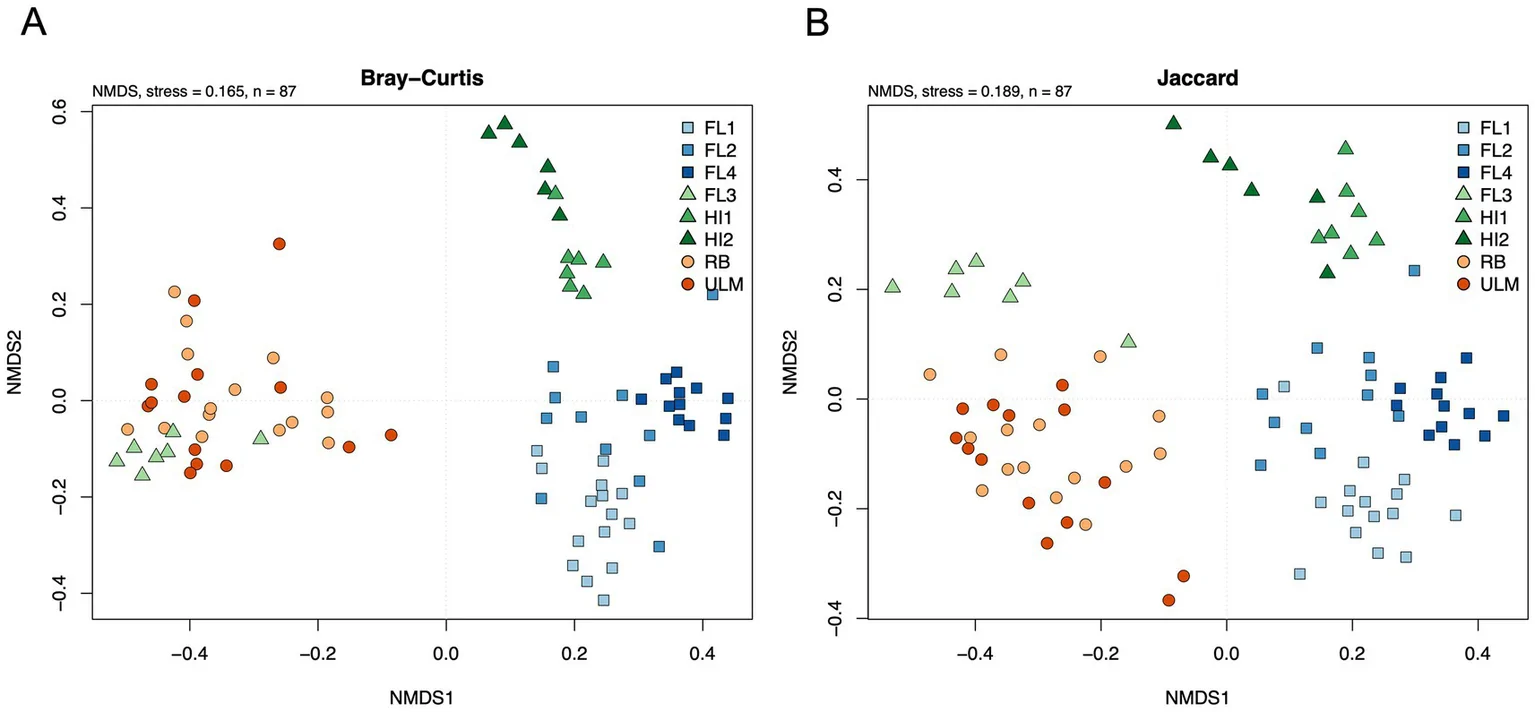
Beta diversity of the common bottlenose dolphin skin microbiome (*Tursiops truncatus*, *n* = 87) across habitat types and facilities. Non-metric multidimensional scaling (NMDS) of Bray-Curtis dissimilarities (**A**; stress = 0.165) and Jaccard distances (**B**; stress = 0.189), calculated on the rarefied feature table. Each point represents one sample. Point shapes denote habitat type: chlorinated tank (squares), enclosed lagoon (triangles) and open bay (circles). Colors indicate individual facilities. Composition differed significantly among habitat types (PERMANOVA: Bray-Curtis *R*^2^ = 0.19, Jaccard *R*^2^ = 0.09; both *p* < 0.001) and among facilities (Bray-Curtis *R*^2^ = 0.41, Jaccard *R*^2^ = 0.21; both *p* < 0.001).

The skin microbiome of dolphins in chlorinated tanks had higher mean relative abundances for 10 out of the 15 bacteria genera containing CRB strains detected in any sample compared to dolphins in enclosed lagoons and open bays (Figure 4; Supplementary Table 4). In contrast to our prediction, dolphins sampled in chlorinated tanks had the lowest mean relative abundance of *Methylobacterium* (Supplementary Table 4). Dolphins in open bays had higher relative abundance and prevalence of *Pseudomonas* than dolphins in enclosed lagoons, while *Vibrio* had higher mean relative abundance in enclosed lagoon dolphins, followed by tank and open bay dolphins (Figure 4; Supplementary Table 4). Five dolphins in open bays tested positive for *Citrobacter* (Supplementary Table 4). Differences in relative abundance between habitat types were statistically tested for seven bacteria genera containing CRB strains (Table 1) with prevalence >30% across the samples. The skin microbiome of dolphins in chlorinated tanks had significantly higher *Staphylococcus* relative abundance (H(2) = 49.09, adj. *p* < 0.001; tank-enclosed lagoon *z* = 4.09, adj. *p* < 0.001; tank-open bay *z* = 6.83, adj. *p* < 0.001) and prevalence (*p* = 0.003; tank-enclosed lagoon adj. *p* = 0.034, tank-open bay adj. *p* = 0.014), supporting our prediction. Significant differences in relative abundance and prevalence were detected for *Legionella*, *Pseudomonas*, *Sphingomonas*, and *Vibrio* (Table 1; Supplementary Table 4). Open bay dolphins had significantly lower *Legionella* relative abundance (H(2) = 9.50, adj. *p* = 0.012; tank-open bay *z* = 2.57, adj. *p* = 0.017; enclosed lagoon-open bay *z* = 2.72, adj. *p* = 0.014) and prevalence (*χ*^2^_2_ = 22.45, *p* < 0.001; tank-open bay adj *p* < 0.001). *Pseudomonas* and *Sphingomonas* were significantly more abundant (*Pseudomonas*: H(2) = 14.92, adj. *p* = 0.001; *Sphingomonas*: H(2) = 11.22, adj. *p* = 0.004) in the skin microbiome of open bay dolphins, in contrast with our prediction. The skin microbiome of enclosed lagoon dolphins had significantly higher *Vibrio* relative abundance (H(2) = 15.38, adj. *p* = 0.001; tank-enclosed lagoon and enclosed lagoon-open bay *z* = 3.48 and *z* = 3.58, adj. *p* = 0.001) and prevalence (*χ*^2^_2_ = 6.36, *p* = 0.042; tank-enclosed lagoon adj. *p* = 0.049, enclosed lagoon-open bay adj. *p* = 0.027). *Acinetobacter* was significantly less prevalent in enclosed lagoon dolphin skin (*χ*^2^_2_ = 11.48, *p* = 0.003; tank-enclosed lagoon adj. *p* = 0.002), while no significant abundance differences were detected. *Halomonas* relative abundance and prevalence did not differ significantly between the three scenarios. Eight bacteria genera containing CRB strains detected on the skin of dolphins in tanks were absent from their enclosure water and six were detected in tank water samples with low mean relative abundance and prevalence (Supplementary Table 5).

**Figure 4:**
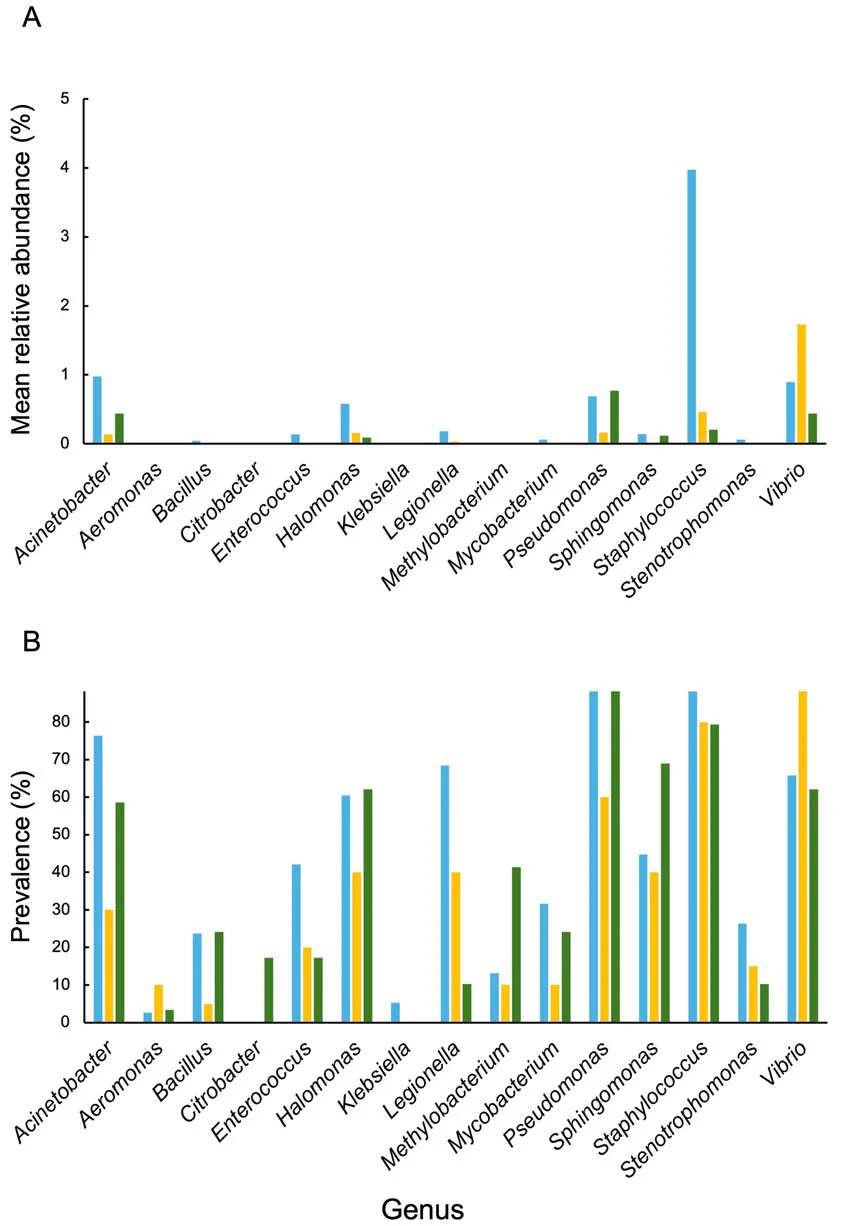
Mean relative abundance and prevalence of chlorine-resistant bacteria genera containing CRB strains in dolphin-associated skin microbiomes for each habitat type. Bars represent: **(A)** mean relative abundance and **(B)** prevalence of bacteria genera containing CRB strains in common bottlenose dolphin (*Tursiops truncatus*) living in chlorinated tanks (*n* = 38; blue), enclosed lagoons (*n* = 20; yellow), and open bays (*n* = 29; green).

**Table 1:** Centered log-ratio (CLR)-transformed count median of bacteria genera containing chlorine-resistant (CRB) strains in the skin microbiome of common bottlenose dolphins (*Tursiops truncatus*) sampled in chlorinated tanks (*n* = 38), enclosed lagoons (*n* = 20), and open bays (*n* = 29).

Genus	Tank	Enclosed lagoon	Open bay	*p-*value
*Acinetobacter*	0.88	−0.81	−0.51	0.150
*Halomonas*	−0.22	−0.75	−0.60	0.649
*Legionella*	−1.06^a^	−0.84^a^	−1.97^b^	0.012
*Pseudomonas*	1.21^a^	0.08^a^	1.80^b^	0.001
*Sphingomonas*	−2.09^a^	−0.82^a^	0.19^b^	0.004
*Staphylococcus*	3.01^a^	1.69^b^	0.84^b^	<0.001
*Vibrio*	0.58^a^	2.07^b^	−0.53^a^	0.001

Kruskal-Wallis Benjamini-Hochberg adjusted (FDR) *p*-value is reported. Superscript letters (a, b) indicate significant differences between habitat types.

### 3.5 Physicochemical effects

Seasonal variations in temperature and pH were consistently recorded in both RB and ULM (Table 2). As expected, salinity changed depending on location and season: RB was polyhaline and salinity increased in Summer; ULM was mostly hypersaline and salinity decreased in Summer (Table 2).

**Table 2:** Temperature, salinity, pH means ± standard error and ranges (brackets) recorded in this study for Redfish Bay (RB) and Upper Laguna Madre (ULM) in Spring and Summer.

Location	Season	Temperature [°C]	Salinity [ppt]	pH
RB	Spring	26.90 ± 0.20 (26.2–27.6)	21.25 ± 0.53 (20–23)	7.85 ± 0.05 (7.72–7.99)
	Summer	31.40 ± 0.23 (30.9–32.4)	26.57 ± 0.37 (25–28)	8.44 ± 0.04 (8.29–8.61)
ULM	Spring	27.0 ± 0.14 (26.8–27.5)	40.6 ± 2.01 (36–45)	8.01 ± 0.04 (7.89–8.12)
	Summer	31.9 ± 0.19 (30.7–32.6)	32.1 ± 1.18 (29–39)	8.73 ± 0.02 (8.64–8.80)

At the phylum level, skin microbiomes differed between specimens collected during Spring and Summer in both RB and ULM, supporting our hypothesis that dolphin skin bacteria communities change seasonally. In RB, Pseudomonadota (r.a._spring_ = 83.6%; r.a._summer_ = 67.6%) and Actinomycetota (r.a._spring_ = 1.3%; r.a._summer_ = 0.9%) were most abundant in Spring, while Bacteroidota (r.a._spring_ = 10.4%; r.a._summer_ = 21.6%) and Bacillota (r.a._spring_ = 0.5%; r.a._summer_ = 5.8%) were most abundant in Summer. In ULM, Bacteroidota (r.a._spring_ = 3.7%; r.a._summer_ = 11.1%) was more abundant in Summer while Actinomycetota (r.a._spring_ = 6.3%; r.a._summer_ = 1.4%) was more abundant in Spring.

Permutation-based stepwise model selection retained salinity (*p* = 0.001) and temperature (*p* = 0.001); pH did not improve the model once temperature was included (*p* = 0.54). The model including all three variables was significant (*p* = 0.001, 999 permutations) suggesting that they influence the skin microbiome composition and beta diversity of common bottlenose dolphins in open bay systems (Figure 5). The first order driver (CCA1) appears to be salinity, showing a spatial pattern from left to right across RB to ULM; the second order factor (CCA2) seems to be a seasonal signal, with Summer samples characterized by high temperature, pH, and salinity in the lower part of the plot, and Spring samples clustering toward the top. Temperature and pH were nearly collinear (VIF = 11.8 and 12.4), whereas salinity was not (VIF = 1.3).

**Figure 5:**
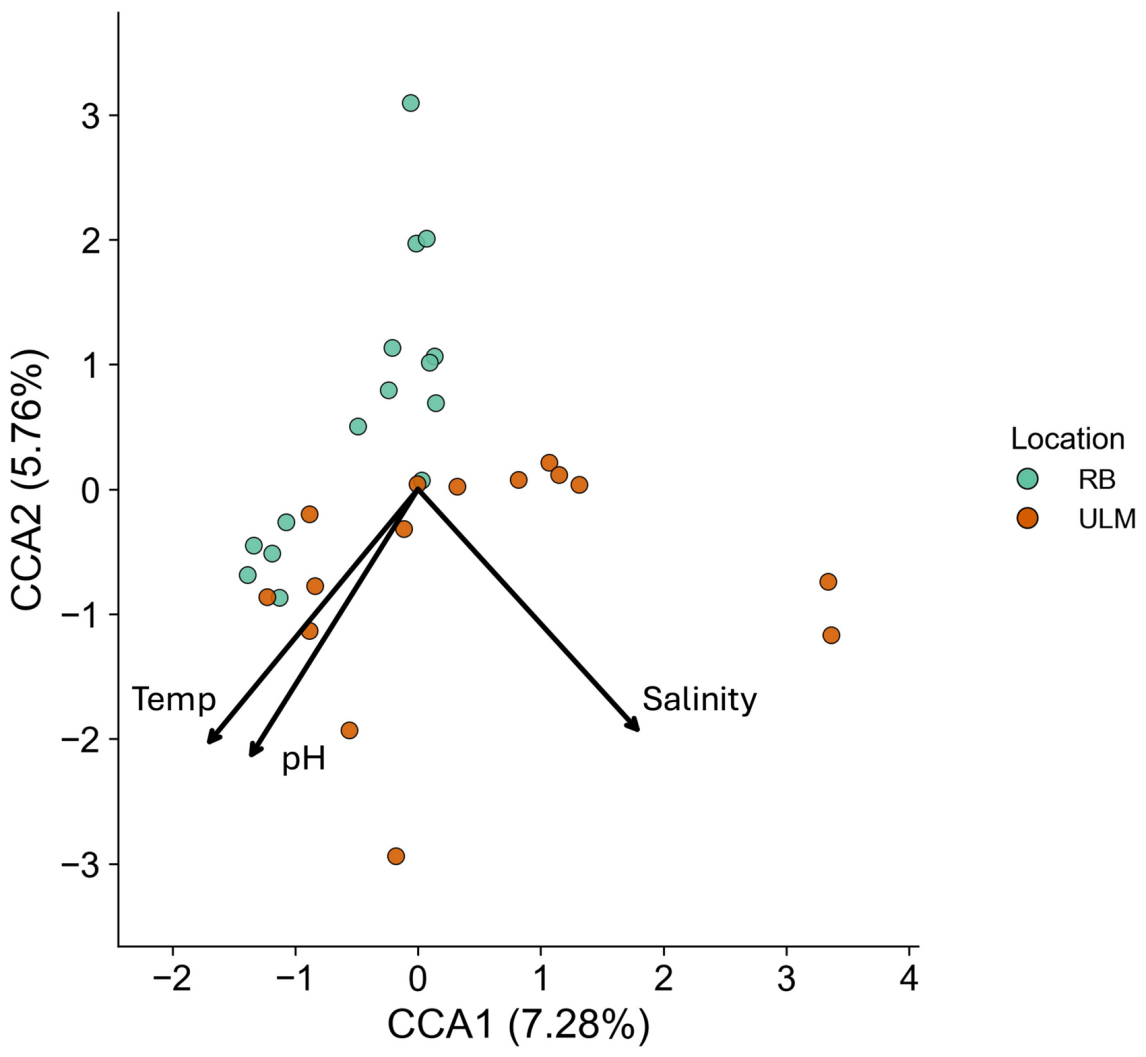
Canonical correspondence analysis (CCA) biplot of dolphin skin microbiome composition from two geographic bays relative to environmental variables. The plot shows skin microbiome patterns for free-swimming common bottlenose dolphins (*Tursiops truncatus*; *n* = 29) living in two open bay systems in Texas, US. Each data point represents one sample. Point colors indicate RB (green) and ULM (orange) open bay systems. Explanatory vectors represent water temperature (Temp), pH, and salinity; arrow length indicates the strength of the variable’s influence on community structure, and arrow direction shows the gradient of increase. The percentages of total inertia (variance) explained by the first two canonical axes (CCA1 and CCA2) are shown in parentheses.

There was support to our prediction that potential opportunistic pathogens associated with slow skin healing in dolphins would be low in relative abundance and prevalence when temperatures and pHs were high. *Mycobacterium*, *Streptococcus* and *Vibrio* were least abundant and prevalent in ULM in Summer (Table 3). In both bays, *Mycobacterium* was least abundant and prevalent in Summer. In RB, *Streptococcus* and *Vibrio* exhibited an opposite pattern increasing their mean relative abundance and prevalence in Summer (Table 3). As *Mycobacterium* prevalence was <30%, relative abundance difference tests were run for *Streptococcus* and *Vibrio* and were not significant. *Streptococcus* prevalence decreased significantly between spring and summer in ULM (*p* = 0.010; ULM spring-ULM summer adj. *p* = 0.018). *Mycobacterium* and *Streptococcus* were not detected in the water samples from open bays. *Vibrio* was present in all water samples from open bays with decreasing proportional shifts in relative abundance from Spring to Summer (RB: r.a._spring_ = 0.2016%, r.a._summer_ = 0.0417%; ULM: r.a._spring_ = 0.1835%; r.a._summer_ = 0.0409%).

**Table 3:** Number of samples (*n*), mean of relative abundance (r.a.) ± standard deviation (s.d.) and prevalence (*p*) of bacteria genera associated with slow healing wounds in the skin microbiome of common bottlenose dolphins (*Tursiops truncatus*) sampled in polyhaline Redfish Bay (RB) and hypersaline Upper Laguna Madre (ULM) in Spring and Summer.

Genus	RB spring (*n* = 8)		RB summer (*n* = 7)	
	r.a. (%) ± s.d.	*p* (%)	r.a. (%) ± s.d	*p* (%)
*Mycobacterium*	0.0342 ± 0.0538	50.0	0.0034 ± 0.0090	14.3
Streptococcus^*^	0.0215 ± 0.0487	37.5^a^	0.0915 ± 0.1902	57.1^ab^
*Vibrio*	0.0967 ± 0.1526	62.5	1.3733 ± 3.3206	85.7

	ULM spring (*n* = 5)		ULM summer (*n* = 9)	
*Mycobacterium*	0.0036 ± 0.0082	20.0	0.0007 ± 0.0022	11.1
Streptococcus^*^	0.2972 ± 0.5084	100.0*^a^*	0.0144 ± 0.0431	11.1^b^
*Vibrio*	0.448 ± 0.6384	80.0	0.0070 ± 0.0116	33.3

(*) denotes significant differences in genera prevalence. Superscript letters (a, b) indicate significant differences between location and season combinations.

## 4 Discussion

Dolphin skin microbiome composition was associated with species and environmental factors including habitat chlorination, seasonality, and salinity. Consistent with previous research (Apprill et al., 2020), closely related dolphin species had distinct skin bacteria communities. Significant increase of skin microbiome evenness, proportional decrease of relative abundance and prevalence of the bacteria genus *Enhydrobacter*, and significant increase of *Staphylococcus* relative abundance and prevalence were associated with habitat chlorination. Our skin microbiome comparison between a large number of common bottlenose dolphins living in tanks, enclosed lagoons, and open bays is unprecedented and highlights how physical and chemical properties of water may impact dolphin health, with important implications for animal husbandry and conservation. Identifying dominant skin commensal bacteria and potential opportunistic pathogens provides biomarkers for health assessment (Ross et al., 2019) and help the formulation on topical probiotics that can improve dolphin care in human-managed facilities and the success of rehabilitation-release conservation programs (Kueneman et al., 2016). Our microbiome data support the low rate of wounds observed on dolphins and fast healing rate during natural exposure to high temperature/pH conditions (Hurst and Orbach, 2022; Guinn et al., 2024).

This study complements the scarce research on rough-toothed dolphin skin microbiome that was limited to four biopsied wild animals (Apprill et al., 2020). Skin microbiome composition differed between rough-toothed and common bottlenose dolphins exposed to the same recirculating water in chlorinated tanks, as documented among other dolphin species in human-management (Chiarello et al., 2017) and in the wild (Apprill et al., 2020). The rough-toothed dolphins in this study were segregated from common bottlenose dolphins housed in the same facility, preventing skin bacteria exchange between dolphin species through physical contact. Although diet influences skin microbiome composition in vertebrates (Ross et al., 2019), both dolphin species in the facility were fed the same food (capelin, *Mallotus villosus*; herring, *Clupea* spp.; lake smelt, *Osmerus mordax*; menhaden*, Brevoortia tyrannus*; squid, *Loligo* spp.). Beluga whales have distinct skin bacterial communities based on sex, potentially reflecting habitat preferences (Van Cise et al., 2020). The present study was limited by a sexually biased, low sample size, with the microbiome of rough-toothed dolphins limited to four females.

Dolphin skin bacteria communities were profoundly distinct from the surrounding water samples, consistent with research on dolphins, beluga whales, and porpoises (Chiarello et al., 2017; Robles-Malagamba et al., 2020; Van Bonn et al., 2022; Zhang, X. et al., 2022). Dolphin skin specimens clustered by habitat type and facility, with dolphins in enclosed lagoons and open bays having a more similar microbiome composition compared to dolphins in chlorinated tanks. *Enhydrobacter* was rare (mean r.a. = 0.9%) on the skin of dolphins living in tanks yet dominant on the skin of dolphins in the other habitat types. *Enhydrobacter* was amongst the most abundant bacteria genera on the skin of common bottlenose dolphins sampled during capture-release programs in Sarasota Bay, Florida (Robles-Malagamba et al., 2020), and in common bottlenose dolphins and killer whales (*Orcinus orca*) in human management (Chiarello et al., 2017). While the function of *Enhydrobacter* in aquatic vertebrate skin microbiomes remains unknown, its commensal nature and resistance to polycyclic aromatic hydrocarbons may be beneficial to outcompete potential pathogens, especially when the host is exposed to pollutants (Sylvain et al., 2020; Hu et al., 2021). Given the lack of functional understanding, *Enhydrobacter*’s benefical role in dolphin skin health remains to be tested. Microbiome samples from dolphins in human management (tanks and enclosed lagoons) clustered by facility. Sharing water, air, diet, and frequent conspecific contact likely promote skin microbiome similarity within a facility, highlighting the strong effect of shared husbandry on skin microbiome composition (Cardona et al., 2018). However, dispersion was lower in open bays and free-swimming dolphins were not more variable among individuals than those in human care.

In contrast to our prediction based on a study on human skin microbiome (Morss-Walton et al., 2022), common bottlenose dolphins living in chlorinated tanks had similar skin microbiome richness and significantly higher evenness compared to conspecifics in enclosed lagoons and open bays. Dolphins completely replace their outermost skin layer every 2–3 h, 8.5× faster than humans (Menon et al., 2022). As skin turns over, it undergoes continuous bacterial decolonization and colonization (Grice and Segre, 2011). Chlorine reduces the microorganism load of water (Gomes et al., 2016) and inhibits the growth of commensal bacteria that would be dominant on dolphin skin in non-chlorinated environments (Robles-Malagamba et al., 2020). The free chlorine in the tanks used in this study was kept at a maximum of 0.8 ppm, about one third of the concentration of swimming pools used by humans (Borgmann-Strahsen, 2003), potentially mitigating chlorination disturbance on dolphin skin microbiome. The high skin microbiome diversity of dolphins in chlorinated tanks may reflect continuous, intermediate epithelial microcosm perturbations (i.e., frequent skin turnover, chlorine bactericide action).

The skin microbiome of dolphins inhabiting chlorinated tanks was associated with a higher mean relative abundance of bacteria genera containing CRB strains compared to the other habitat types, although proportional shifts in relative abundance could not be statistically verified for all 15 genera considered in this study. *Staphylococcus* relative abundance and prevalence were significantly higher in tank dolphins, supporting our prediction. In contrast, *Pseudomonas* and *Sphingomonas* had significantly higher relative abundance in open bay dolphins, while *Vibrio* was highest in enclosed lagoon dolphins. *Legionella* had significantly lower relative abundance in open bay dolphins, yet no significant differences were detected between tank and enclosed lagoon dolphins. While our results may have been confounded by untested variables (e.g., animal density, social contact frequency, water turnover rate) and the uneven sample size across habitat types, several bacteria genera may include both chlorine-sensitive and CRB strains that could not be discriminated by 16S rRNA amplicon data. Proportional shifts in relative abundance should not be interpreted as evidence of changes in absolute abundances. In future studies, absolute quantification methods, such as qPCR (Wu et al., 2014) and spiking in internal controls (Lin et al., 2019), would be required to determine whether CRB strains actually proliferate under chlorinated conditions. Bacteria genera usually found in dolphin skin microbiome (e.g., *Enhydrobacter, Tenacibaculum*) (Robles-Malagamba et al., 2020) did not dominate the bacterial communities of dolphins in chlorinated tanks. Bacteria can survive water chlorination by forming biofilms on tank surfaces (Luo et al., 2021; Wang et al., 2023). CRB strains likely colonize dolphin skin by direct contact with biofilms adhering to surfaces and/or other dolphins. Free-swimming Indo-Pacific bottlenose dolphins rub against coral and sponges to self-medicate skin (Morlock et al., 2022). Self-rubbing against objects (e.g., enclosure bottom, netting, floating objects) occurs often among human-managed cetaceans (Dudzinski et al., 2012). While dolphin interactions with the walls of their enclosures may promote CRB strain skin colonization, recruitment from habitat water may be limited to occasional fluctuations of free chlorine concentration supporting bacteria survival (Borgmann-Strahsen, 2003). Most of the genera containing CRB strains detected in dolphin skin samples were absent in habitat water. However, *Halomonas, Legionella, Pseudomonas, Sphingomonas, Staphylococcus*, and *Vibrio* were identified in low relative abundance and prevalence, possibly because they reside in biofilms rather than in the water column (Luo et al., 2021; Wang et al., 2023). The high relative abundance of potential opportunistic pathogens in the skin samples from dolphins inhabiting chlorinated tanks may indicate health trade-offs. *Staphylococcus delphini* has been associated with skin infections in common bottlenose dolphins (Romani-Cremaschi et al., 2023). *Vibrio* spp. are opportunist pathogens causing slow healing ulcers in melon-headed whales (*Peponocephala electra*) (Li et al., 2025). *Enterococcus* and *Legionella* are not usually associated with cetacean skin microbiomes but some strains can cause severe infections in other body sites (Chitasombat et al., 2018; Fan et al., 2024). As chlorine-resistance and pathogenicity can vary substantially among species and strains within the same genus (Zhang, Z. et al., 2022; Wang et al., 2023), isolation and identification of the specific strains colonizing dolphin skin in chlorinated waters is necessary to assess pathogenic risk. Consensus on chlorine-resistance definition and evaluation methods differs in literature (Luo et al., 2021), and our comparison may not have been comprehensive of all bacteria genera containing CRB strains. The facilities included in our comparison had tanks with similar temperature, salinity, and pH. Future studies of dolphin skin microbiome could explore temporal variations of CRB strain relative abundance and prevalence across gradients of environmental factors in chlorinated aquaria.

The skin microbiome composition of free-swimming common bottlenose dolphins appeared to be influenced by habitat temperature, salinity, and pH. Temperature and pH were highly correlated and fluctuated seasonally, posing challenges differentiating their impacts. Temperature variations impaired core bacteria dominance in the skin microbiome of humpback whales (Bierlich et al., 2018) and killer whales (Hooper et al., 2019), although pH was not assessed and its effect on cetacean skin microbiome is unknown. Seasonal differences of bacteria community composition at the phylum level were slightly distinct between RB and ULM, suggesting other factors shaped dolphin skin microbiome. RB is a polyhaline habitat characterized by seasonal changes in salinity (22–35 ppt; 3-year arithmetic mean = 28.47 ppt), while ULM is a hypersaline lagoon with salinity often exceeding 45 ppt (3-year arithmetic mean = 35.67 ppt) (Powers and Wetz, 2025). Our environmental measurements support these trends. Despite consistent temperature and pH levels between the two open bay systems, dolphins in each bay had distinct microbiomes, potentially driven by salinity gradients. Geographic-driven skin microbiome differentiation has been documented in beluga whales inhabiting two neighboring bays in Alaska, US (Van Cise et al., 2020), and may reflect a combination of environmental parameters.

As predicted, dolphins sampled in ULM waters in summer were associated with the lowest *Mycobacterium*, *Streptococcus*, and *Vibrio* mean relative abundance and prevalence, potentially explaining the fast skin wound healing rate documented in dolphins inhabiting ULM (Hurst and Orbach, 2022). While pathogenicity is typically species or even strain specific, several *Mycobacterium*, *Streptococcus*, and *Vibrio* species occur in necrotic and ulcerated cutaneous wounds of dolphins (Souter et al., 2021, 2026; Li et al., 2025). The low relative abundance and prevalence of *Mycobacterium* on dolphins biopsied in Summer in both bay systems suggest that high temperatures (30.7–32.6 °C), salinities (>25 ppt), and pHs (8.29–8.80) may impede this bacteria’s growth on cetacean epithelium. Most aquatic *Mycobacterium* species thrive in the range of temperatures measured in RB and ULM in Spring and Summer (Torvinen et al., 2007), although their growth is optimal at low salinity (Jacobs et al., 2009) and acidic pH (Bal Krishna et al., 2024). The higher temperature and more basic pH measured in summer coincided with an approximately 10-fold decrease in the mean relative abundance of *Mycobacterium*. However, this pattern could not be statistically verified because *Mycobacterium* prevalence was < 30% across all samples. Given *Mycobacterium* associations with ulcerating skin lesions in bottlenose dolphin species (Souter et al., 2026), unfavorable physical and chemical conditions for growth may facilitate healing processes, potentially explaining the low wound rate observed in Summer (Guinn et al., 2024). The increase of *Streptococcus* and *Vibrio* on the skin of dolphins sampled in RB during the Summer may reflect differences in nutrient loads between RB and ULM, as well as a higher tolerance for temperature and pH fluctuations. While ULM nitrate levels have been decreasing in the last 3 years, RB is experiencing an opposite trend and is showing signs of water quality degradation, potentially as it receives stormwater runoff and wastewater treatment plant effluents from the neighboring residential areas (Powers and Wetz, 2025). *Vibrio* and marine-adapted *Streptococcus* species can survive a wide range of temperatures and pHs (Restrepo et al., 2005; Velez et al., 2023; Numberger et al., 2024), similar to those recorded in this study, and may outcompete less tolerant skin colonizers, especially when Summer warmer waters promote an even more frequent skin turnover (Russo et al., 2018).

Several study limitations warrant further assessment. Human-cared and free-swimming dolphins were sampled on different areas of their body (i.e., ventral swab vs. dorsal biopsy swab). Despite a relatively large sample size for common bottlenose dolphins compared to previous studies, unbalanced sampling across habitat types due to the opportunistic nature of the study precluded a robust statistical analysis for some of the bacteria genera. Potential confounding variables (e.g., animal density, social contact frequency, water turnover rate, demography of open bay dolphins) hampered a direct causal inference about environmental effects on dolphin skin microbiome diversity and composition. Our 16S rRNA microbiome characterization limited taxonomic resolution to the genus-level, yet chlorine-resistance and pathogenicity may be strain-specific and require culture-based and genomic methods to ascertain certainty.

The chlorination and high temperature/pH associations with significant changes of microbiome diversity and composition in common bottlenose dolphin skin highlight an important signal with conservation and animal husbandry implications. Bacteria genera containing potentially opportunistic pathogens may be favored in human-managed and wild habitats by environmental parameter modifications. The dominance of *Enhydrobacter* on dolphins in enclosed lagoons and open bays yet low relative abundance in tanks solicits the need for metagenomic analyses, culture-based *in vitro* antagonistic assays, and ex vivo biopsy analysis of functionality in dolphin skin microbiome. Future studies incorporating co-occurrence network could provide further insight into potential microbial interactions and community stability across dolphin habitats. The significant increase of *Staphylococcus* relative abundance and prevalence in dolphins inhabiting tanks warrants culture-based investigation to identify strains, verify chlorine-resistance, and track colonization patterns. Future research could focus on human-cared dolphins housed in habitats with similar environmental parameters and husbandry practices to reduce the number of potentially confounding variables and ascertain causal relationships based on the association detected in the present study.

## Data Availability

The data analyzed in this study are available in the NCBI SRA (https://www.ncbi.nlm.nih.gov/sra) under BioProject accession PRJNA1492278.
