# Supplementary material for: Water chlorination and environmental factors are associated with dolphin skin microbiome diversity and composition
Source: Front Microbiol. 2026 Oct 28;17:1934395. doi: 10.3389/fmicb.2026.1934395 (PMC13753770; doi:10.3389/fmicb.2026.1934395)
Supplement: Supplementary file 1 [file Image_1.PDF]

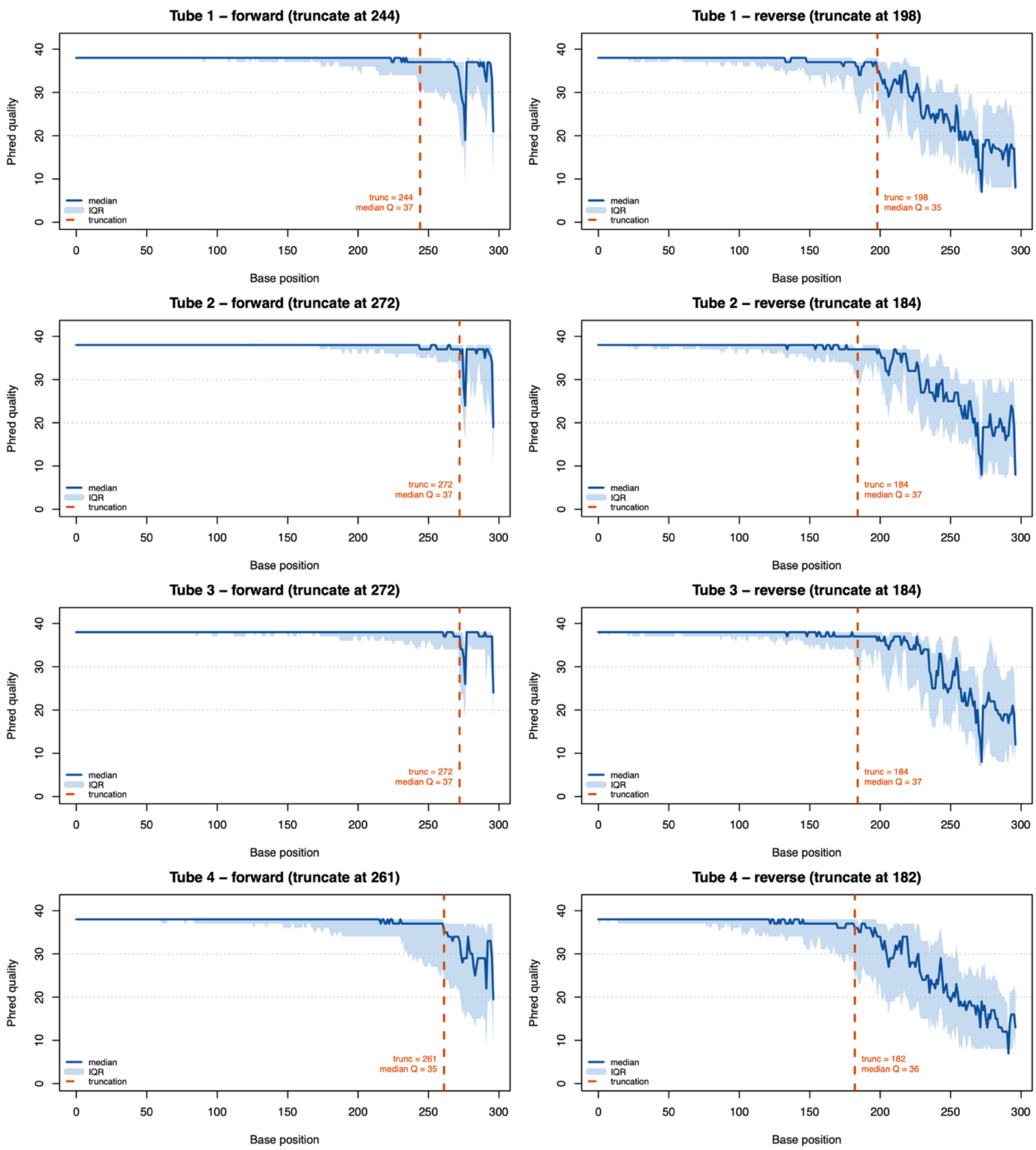

Supplementary Figure 1. Per-run sequence quality profiles and DADA2 truncation positions. Median Phred quality score (dark blue line) and interquartile range (shaded band) at each base position for forward (left) and reverse (right) reads of each of the four MiSeq runs, generated from the demultiplexed reads after primer removal. The dashed vertical line marks the truncation position applied to that run, annotated with the median quality score at that position.
