# Supplementary material for: Water chlorination and environmental factors are associated with dolphin skin microbiome diversity and composition
Source: Front Microbiol. 2026 Oct 28;17:1934395. doi: 10.3389/fmicb.2026.1934395 (PMC13753770; doi:10.3389/fmicb.2026.1934395)
Supplement: Supplementary file 2 [file Image_2.PDF]

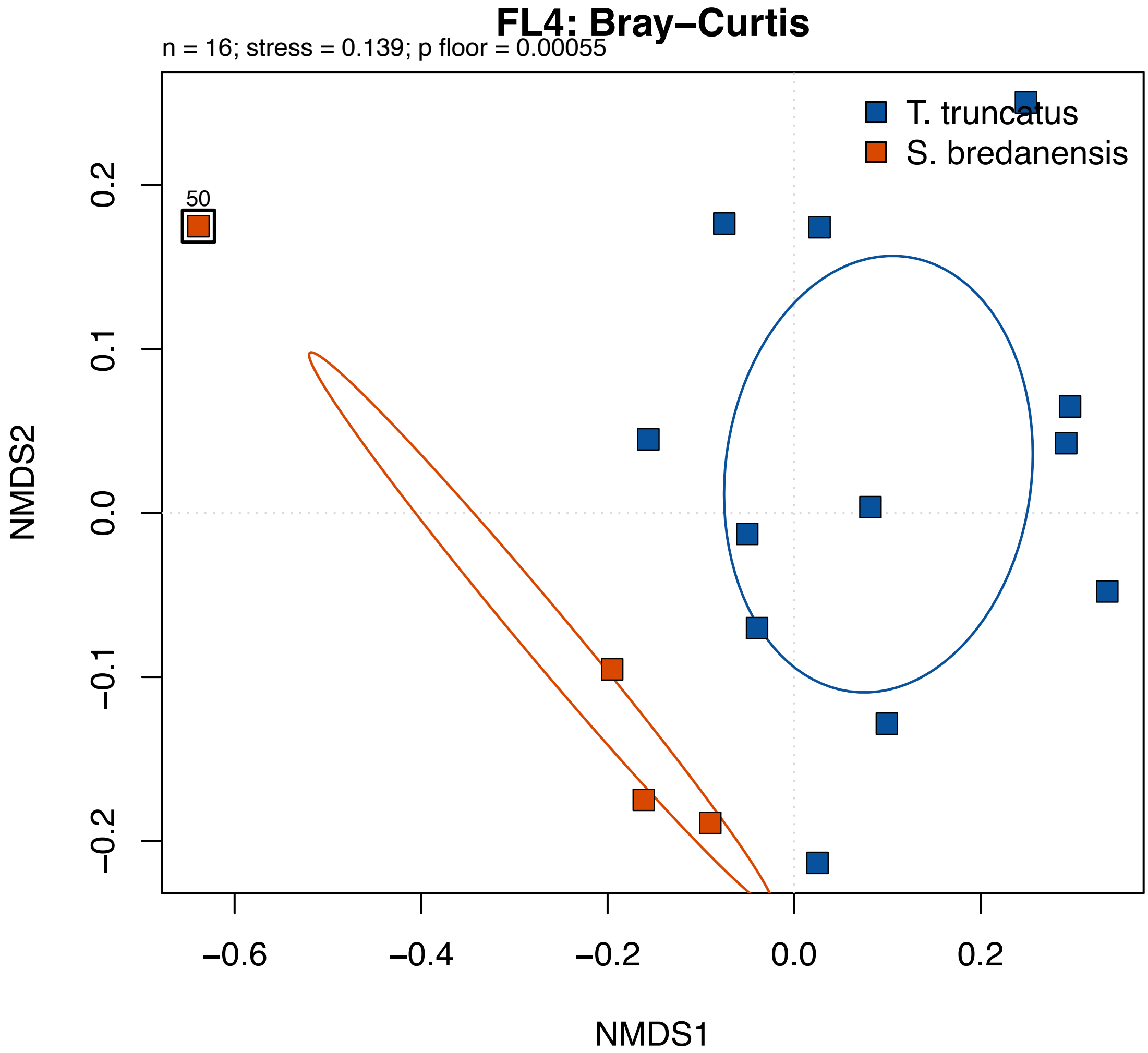

Supplementary Figure 2. Skin microbiome composition of common bottlenose dolphins (*Tursiops truncatus*, n = 12) and rough-toothed dolphins (*Steno bredanensis*, n = 4) housed at the same facility (FL4). Non-metric multidimensional scaling of Bray-Curtis dissimilarities calculated on the rarefied feature table (stress = 0.139). Ellipses indicate one standard deviation around each group centroid. The outlined symbol labelled 50 identifies the rough-toothed dolphin whose skin community was dominated by two *Vibrio* ASVs. Composition differed significantly between species (PERMANOVA $R^2 = 0.14$, $p = 0.004$) with homogeneous dispersion (PERMDISP $p = 0.147$).
