# Supplementary material for: Water chlorination and environmental factors are associated with dolphin skin microbiome diversity and composition
Source: Front Microbiol. 2026 Oct 28;17:1934395. doi: 10.3389/fmicb.2026.1934395 (PMC13753770; doi:10.3389/fmicb.2026.1934395)
Supplement: Supplementary file 3 [file Image_3.PDF]

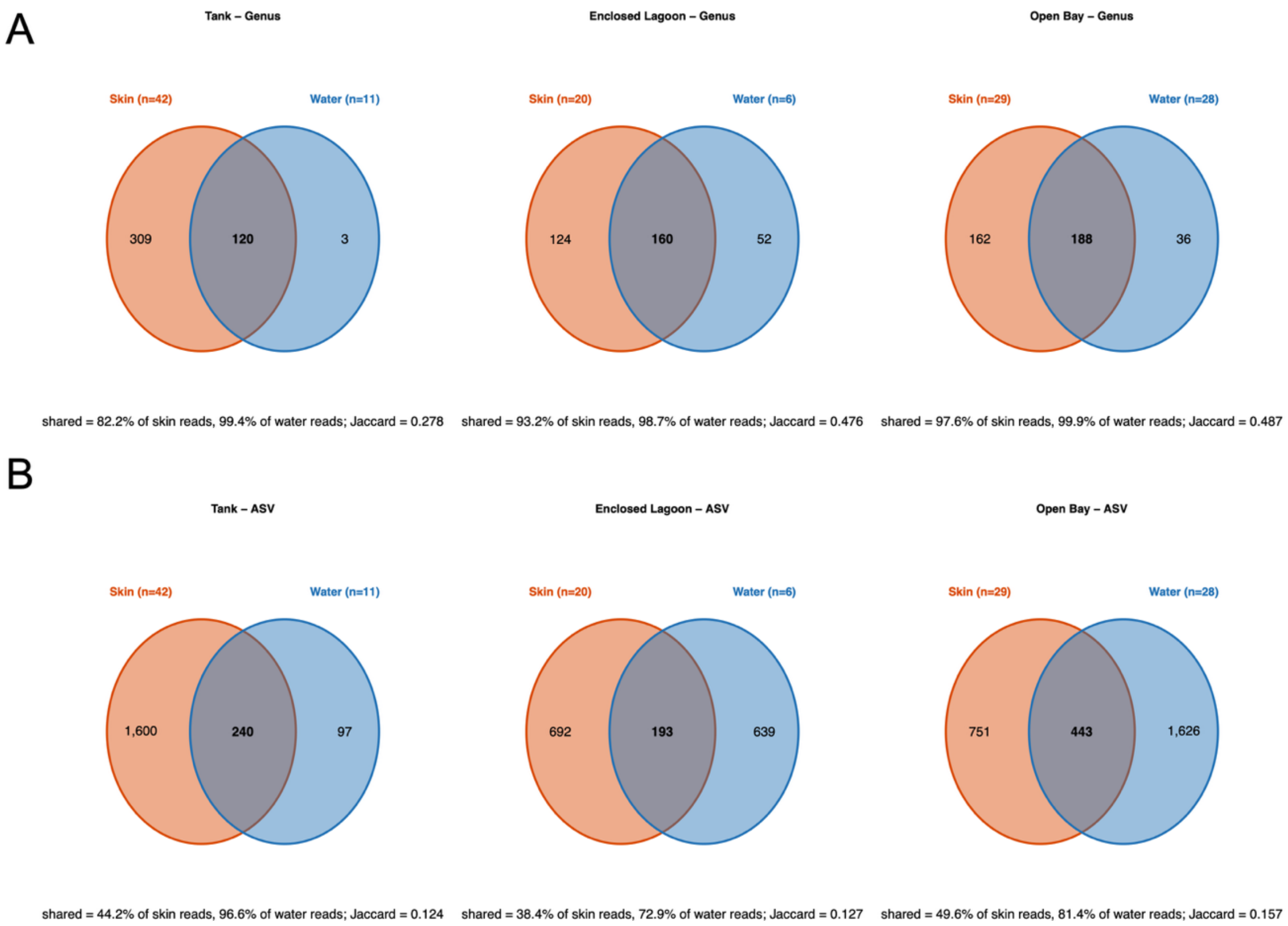

Supplementary Figure 3. Overlap between dolphin skin and surrounding water bacterial communities, by habitat type. Venn diagrams show taxa detected in skin swabs (orange) and water samples (blue) from chlorinated tanks, enclosed lagoons and open bays, at genus level (A) and sequence-variant level (B). A taxon was counted as present when detected in at least two samples of a given type. Numbers within each region give taxon counts; text below each panel gives the proportion of skin and of water reads belonging to shared taxa, and the Jaccard similarity of the two taxon lists. Circle areas are not proportional to taxon counts.
