# Supplementary material for: Water chlorination and environmental factors are associated with dolphin skin microbiome diversity and composition
Source: Front Microbiol. 2026 Oct 28;17:1934395. doi: 10.3389/fmicb.2026.1934395 (PMC13753770; doi:10.3389/fmicb.2026.1934395)
Supplement: Supplementary file 4 [file Table_1.PDF]

Supplementary Table 1. Common bottlenose dolphins (*Tursiops truncatus*, Tt) and rough-toothed dolphins (*Steno bredanensis*, Sb) from chlorinated aquaria (tank) included in the study.

Sample ID	Species	Facility	Sex	Age	Years in facility	T[°C]	Salinity[ppt]	pH
1	Tt	FL1	f	15	15	25.0	33	7.8
2	Tt	FL1	f	56	51	25.0	33	7.8
3	Tt	FL1	m	20	20	25.0	33	7.8
4	Tt	FL1	f	22	22	25.0	33	7.8
5	Tt	FL1	f	35	35	25.0	33	7.8
6	Tt	FL1	f	15	15	25.0	33	7.8
7	Tt	FL1	m	46	34	25.0	33	7.8
8	Tt	FL1	m	13	13	25.0	33	7.8
9	Tt	FL1	m	30	30	25.0	33	7.8
10	Tt	FL1	m	8	8	25.0	33	7.8
11	Tt	FL1	f	39	35	25.0	33	7.8
12	Tt	FL1	m	40	40	25.0	33	7.8
13	Tt	FL1	m	8	8	25.0	33	7.8
14	Tt	FL1	f	16	16	25.0	33	7.8
15	Tt	FL1	m	29	29	25.0	33	7.8
16	Tt	FL2	f	30	30	23.0	35	7.5
17	Tt	FL2	f	10	10	23.0	35	7.5
18	Tt	FL2	f	10	10	23.0	35	7.5
19	Tt	FL2	f	25	25	23.0	35	7.5
20	Tt	FL2	f	14	14	23.0	35	7.5
21	Tt	FL2	f	25	25	23.0	35	7.5
22	Tt	FL2	f	27	27	23.0	35	7.5
23	Tt	FL2	f	25	25	23.0	35	7.5
24	Tt	FL2	f	38	38	23.0	35	7.6
25	Tt	FL2	m	13	13	23.0	35	7.5
26	Tt	FL2	m	24	24	23.0	35	7.5
27	Tt	FL2	m	48	48	23.0	35	7.5
35	Tt	FL4	m	18	18	25.0	35	7.4
36	Tt	FL4	f	10	10	25.0	35	7.4
37	Tt	FL4	m	18	19	25.0	35	7.4
38	Tt	FL4	m	14	14	25.0	35	7.4
39	Tt	FL4	m	13	13	25.0	35	7.4
40	Tt	FL4	f	18	18	25.0	35	7.4
41	Tt	FL4	m	19	19	25.0	35	7.4
42	Tt	FL4	f	7	7	25.0	35	7.4
43	Tt	FL4	f	38	35	25.0	35	7.4
44	Tt	FL4	f	7	7	25.0	35	7.4
45	Tt	FL4	m	13	13	25.0	35	7.4
46	Tt	FL4	m	14	14	25.0	35	7.4
47	Sb	FL4	f	8	6	25.0	35	7.4
48	Sb	FL4	f	27	20	25.0	35	7.4
49	Sb	FL4	f	16	15	25.0	35	7.4
50	Sb	FL4	f	4	1	25.0	36	7.4
