# Supplementary material for: Water chlorination and environmental factors are associated with dolphin skin microbiome diversity and composition
Source: Front Microbiol. 2026 Oct 28;17:1934395. doi: 10.3389/fmicb.2026.1934395 (PMC13753770; doi:10.3389/fmicb.2026.1934395)
Supplement: Supplementary file 5 [file Table_2.PDF]

Supplementary Table 2. Common bottlenose dolphins (*Tursiops truncatus*) from enclosed lagoons included in the study.

Sample ID	Facility	Sex	Age	Human care time	T[°C]	Salinity[ppt]	pH
28	FL3	m	41	35	21.7	-	8.3
29	FL3	f	23	23	21.7	-	8.3
30	FL3	m	21	21	21.7	-	8.3
31	FL3	m	20	20	21.7	-	8.3
32	FL3	f	19	19	21.7	-	8.3
33	FL3	m	15	12	21.7	-	8.3
34	FL3	m	9	9	21.7	-	8.3
51	HI2	m	39	36	26.7	24	7.7
52	HI2	m	27	27	26.7	24	7.7
53	HI2	m	30	30	26.7	24	7.7
54	HI2	m	26	16	26.7	24	7.7
55	HI2	m	33	33	27.2	25	7.8
56	HI2	m	29	29	27.2	25	7.8
58	HI1	f	39	36	25.5	30	8.3
59	HI1	f	5	5	25.5	30	8.3
60	HI1	f	17	17	25.5	30	8.3
61	HI1	f	24	24	25.5	30	8.3
62	HI1	m	25	25	25.5	30	8.3
63	HI1	f	14	14	25.5	30	8.3
64	HI1	f	4	4	25.5	30	8.3
