# Supplementary material for: Water chlorination and environmental factors are associated with dolphin skin microbiome diversity and composition
Source: Front Microbiol. 2026 Oct 28;17:1934395. doi: 10.3389/fmicb.2026.1934395 (PMC13753770; doi:10.3389/fmicb.2026.1934395)
Supplement: Supplementary file 6 [file Table_3.PDF]

Supplementary Table 3. Common bottlenose dolphins (*Tursiops truncatus*) biopsied in Texas open bay systems included in the study.

Sample ID	Bay	Season	T[°C]	Salinity[ppt]	pH
1B	RB	Spring	26.9	21	7.63
2B	ULM	Spring	26.8	45	7.89
3B	ULM	Spring	26.8	41	8.01
4B	ULM	Spring	26.8	36	8.1
5B	ULM	Spring	26.9	36	8.12
6B	ULM	Spring	27.5	45	7.95
7B	RB	Spring	26.2	20	7.72
8B	RB	Spring	26.2	20	7.72
9B	RB	Spring	26.4	23	7.87
10B	RB	Spring	27.5	23	7.96
11B	RB	Spring	27.6	23	7.98
12B	RB	Spring	26.8	20	7.93
13B	RB	Spring	27.3	20	7.99
14B	RB	Summer	30.9	25	8.29
15B	RB	Summer	30.8	27	8.39
16B	RB	Summer	30.8	27	8.39
17B	RB	Summer	31.8	26	8.4
18B	ULM	Summer	30.7	39	8.64
19B	ULM	Summer	32.1	30	8.77
20B	ULM	Summer	32.1	30	8.77
21B	ULM	Summer	32.2	29	8.78
22B	ULM	Summer	32.4	33	8.74
23B	ULM	Summer	32.6	37	8.74
32B	ULM	Summer	31.4	30	8.63
33B	ULM	Summer	31.8	30	8.72
34B	ULM	Summer	32.2	31	8.78
35B	ULM	Summer	31.8	35	8.8
37B	RB	Summer	31.7	28	8.46
38B	RB	Summer	32.4	26	8.61
39B	RB	Summer	31.5	27	8.56
