# Supplementary material for: Water chlorination and environmental factors are associated with dolphin skin microbiome diversity and composition
Source: Front Microbiol. 2026 Oct 28;17:1934395. doi: 10.3389/fmicb.2026.1934395 (PMC13753770; doi:10.3389/fmicb.2026.1934395)
Supplement: Supplementary file 7 [file Table_4.PDF]

Supplementary Table 4. Mean relative abundance (r.a.) ± standard deviation (s.d.) and prevalence (p) of chlorine-resistant bacteria (CRB) genera in the skin microbiome of common bottlenose dolphins (*Tursiops truncatus*) sampled in chlorinated tanks (n =38), enclosed lagoons (n = 20), and open bays (n = 29). (*) denotes genera with significant (Chi-square or Fisher-Freeman-Halton exact tests, $p < 0.05$) difference in prevalence across the habitat types. Superscript letters (*a*, *b*) indicate significant differences (Fisher's exact test, Bonferroni adjusted $p < 0.166$) between habitat types.

	**Tank**		**Enclosed lagoon**		**Open bay**	
**Genus**	**r.a. ± s.d. (%)**	**p (%)**	**r.a. ± s.d. (%)**	**p (%)**	**r.a. ± s.d. (%)**	**p (%)**
*Acinetobacter**	0.9783 ± 2.1955	76.3 [a]	0.1343 ± 0.4940	30.0 [b]	0.4403 ± 1.5891	58.6 [a]
Aeromonas	0.0032 ± 0.0194	2.6	0.0054 ± 0.0171	10.0	0.0008 ± 0.0045	3.4
Bacillus	0.0465 ± 0.2096	23.7	0.0002 ± 0.0008	5.0	0.0132 ± 0.0391	24.1
*Citrobacter**	-	- [a]	-	- [a]	0.0025 ± 0.0076	17.2 [b]
Enterococcus	0.1388 ± 0.4289	42.1	0.0122 ± 0.0416	20.0	0.0060 ± 0.0224	17.2
Halomonas	0.5824 ± 1.9109	60.5.9	0.0162 ± 0.0271	40.0	0.0911 ± 0.2068	62.1
Klebsiella	0.0120 ± 0.0647	5.3	-	-	-	-
*Legionella**	0.1804 ± 0.2935	68.4 [a]	0.0347 ± 0.0972	40.0 [a]	0.0055 ± 0.0180	10.3 [b]
*Methylobacterium**	0.0048 ± 0.0157	13.2 [a]	0.0052 ± 0.0179	10.0 [a]	0.0112 ± 0.0181	41.4 [b]
Mycobacterium	0.0625 ± 0.1872	31.6	0.0115 ± 0.0355	10.0	0.0111 ± 0.0311	24.1
*Pseudomonas**	0.6921 ± 0. 9201	89.5 [a]	0.1630 ± 0.3570	60.0 [b]	0.7724 ± 1.5857	93.1 [a]
Sphingomonas	0.1422 ± 0.2457	44.7	0.0105 ± 0.0205	40.0	0.1214 ± 0.1686	69.0
*Staphylococcus**	3.9748 ± 4.0677	100[a]	0.4618 ± 0.8974	80.0 [b]	0.2072 ± 0.3933	79.3 [b]
Stenotrophomonas	0.0608 ± 0.1627	26.3	0.0013 ± 0.0037	15.0	0.0030 ± 0.0102	10.3
*Vibrio**	0.8988 ± 2.0273	65.8 [a]	1.7339 ± 3.7552	95.0 [b]	0.4377 ± 1.6549	62.1 [a]
