# Supplementary material for: Water chlorination and environmental factors are associated with dolphin skin microbiome diversity and composition
Source: Front Microbiol. 2026 Oct 28;17:1934395. doi: 10.3389/fmicb.2026.1934395 (PMC13753770; doi:10.3389/fmicb.2026.1934395)
Supplement: Supplementary file 8 [file Table_5.PDF]

Supplementary Table 6. Mean relative abundance (r.a.) ± standard deviation (s.d.) and prevalence (p) of chlorine-resistant bacteria (CRB) genera in the the water microbiome of chlorinated tanks (n =11), enclosed lagoons (n = 6), and open bays (n = 28).

	Tank		Enclosed lagoon		Open bay	
**Genus**	**r.a. (%) ± s.d.**	**p (%)**	**r.a. (%) ± s.d.**	**p (%)**	**r.a. (%) ± s.d.**	**p (%)**
Acinetobacter	-	-	0.0041 ± 0.0101	16.7	0.0009 ± 0.0046	3.6
Aeromonas	-	-	0.0033 ± 0.0082	16.7	-	-
Bacillus	-	-	-	-	-	-
Citrobacter	-	-	-	-	-	-
Enterococcus	-	-	-	-	-	-
Halomonas	0.0017 ± 0.0053	10.0	0.0205 ± 0.0227	83.3	0.3277 ± 0.3874	100.0
Klebsiella	-	-	-	-	-	-
Legionella	0.0117 ± 0.0232	30.0	0.0282 ± 0.0481	50.0	0.0092 ± 0.0188	53.6
Methylobacterium	-	-	-	-	-	-
Mycobacterium	-	-	-	-	-	-
Pseudomonas	0.0711 ± 0.1524	50.0	0.6509 ± 1.2552	100.0	3.2959 ± 1.8041	100.0
Sphingomonas	0.0138 ± 0.0438	10.0	0.0043 ± 0.0067	33.3	-	-
Staphylococcus	0.1071 ± 0.3386	10.0	0.0037 ± 0.0048	50.0	0.0027 ± 0.0059	25.0
Stenotrophomonas	-	-	-	-	-	-
Vibrio	0.0330 ± 0.0649	40.0	1.9874 ± 1.0308	100.0	0.1067 ± 0.0960	92.8
